# Targeting pancreatic cancer metabolic dependencies through glutamine antagonism

**DOI:** 10.1038/s43018-023-00647-3

**Published:** 2023-10-09

**Authors:** Joel Encarnación-Rosado, Albert S. W. Sohn, Douglas E. Biancur, Elaine Y. Lin, Victoria Osorio-Vasquez, Tori Rodrick, Diana González-Baerga, Ende Zhao, Yumi Yokoyama, Diane M. Simeone, Drew R. Jones, Seth J. Parker, Robert Wild, Alec C. Kimmelman

**Affiliations:** 1 Perlmutter Cancer Center, New York University Grossman School of Medicine, New York, NY USA; 2 Department of Radiation Oncology, New York University Grossman School of Medicine, New York, NY USA; 3 Division of Advanced Research Technologies, New York University School of Medicine, New York, NY USA; 4 Dracen Pharmaceuticals, Inc., San Diego, CA USA; 5 Department of Biochemistry & Molecular Biology, University of British Columbia, Vancouver, British Columbia Canada

**Keywords:** Cancer metabolism, Pancreatic cancer, Cancer therapy, Cancer

## Abstract

Pancreatic ductal adenocarcinoma (PDAC) cells use glutamine (Gln) to support proliferation and redox balance. Early attempts to inhibit Gln metabolism using glutaminase inhibitors resulted in rapid metabolic reprogramming and therapeutic resistance. Here, we demonstrated that treating PDAC cells with a Gln antagonist, 6-diazo-5-oxo-l-norleucine (DON), led to a metabolic crisis in vitro. In addition, we observed a profound decrease in tumor growth in several in vivo models using sirpiglenastat (DRP-104), a pro-drug version of DON that was designed to circumvent DON-associated toxicity. We found that extracellular signal-regulated kinase (ERK) signaling is increased as a compensatory mechanism. Combinatorial treatment with DRP-104 and trametinib led to a significant increase in survival in a syngeneic model of PDAC. These proof-of-concept studies suggested that broadly targeting Gln metabolism could provide a therapeutic avenue for PDAC. The combination with an ERK signaling pathway inhibitor could further improve the therapeutic outcome.

## Abstract (web summary)

Kimmelman and colleagues show that inhibition of glutamine (Gln) metabolism using a Gln antagonist decreases pancreatic cancer tumor growth, but is compensated by upregulation of extracellular signal-regulated kinase signaling, which can be co-targeted.

## Main

Pancreatic ductal adenocarcinoma (PDAC) has a poor prognosis, with a 5-year overall survival rate of approximately11%^1,2^. A key feature associated with this poor prognosis is the complex tumor microenvironment (TME) in which these tumors develop^3^. The PDAC TME consists of a dense fibrous stroma (desmoplasia) and heterogeneous cellular populations (for example, cancer-associated fibroblasts, neurons)^4^. These factors, along with poor vascular perfusion, reduce nutrient accessibility, promote intratumoral hypoxia and decrease the infiltration of immune cells. Many studies demonstrated that PDAC cells thrive in the austere TME by reprogramming their metabolism and relying on scavenging pathways (for example, autophagy, macropinocytosis)^5^. Despite advances in the past decade regarding fuel source use and the dynamic metabolism of PDAC, we have not yet effectively translated this knowledge into clinically relevant therapeutic interventions.

Glutamine (Gln) has pleiotropic roles in cellular metabolism^6^. For example, the carbon skeleton of Gln contributes to the tricarboxylic acid (TCA) cycle intermediates. This process is mediated by glutaminase (GLS), which converts Gln into glutamate (Glu). Gln-derived Glu is further converted into α-ketoglutarate (AKG) by the activity of Glu dehydrogenase or by transaminase activity (for example, aspartate transaminase, glutamate oxaloacetate transaminase 1 (GOT1) and GOT2). AKG is further used to generate other TCA cycle intermediates, lipids and reducing equivalents^7,8^. The amide nitrogen of Gln supplies nitrogen for the biosynthesis of several metabolites, including amino acids, hexosamines and nucleotides^9^. Previous work from our group demonstrated that PDAC cells preferentially metabolize Gln through the activity of the transamination reactions of GOT1 and GOT2 to maintain nicotinamide adenine dinucleotide phosphate and nicotinamide adenine dinucleotide pools, supporting tumor growth and redox balance^10,11^.

To target this metabolic dependency, we previously tested GLS inhibition in pancreatic cancer models^12^. While GLS inhibition transiently reduced cellular proliferation in vitro, it was ineffective across multiple in vivo PDAC models. Resistance was driven by a global metabolic rewiring, resulting in changes in fuel source use and downstream metabolic fluxes^12^. We hypothesized that inhibiting Gln metabolism more broadly may improve efficacy and prevent the rapid metabolic rewiring that leads to resistance. Using a Gln analog, such as 6-diazo-5-oxo-l-norleucine (DON), which covalently and irreversibly binds to Gln-metabolizing enzymes will broadly inhibit Gln metabolism, in contrast to GLS inhibition, which only blocks the use of Gln-derived Glu. DON specifically inhibits Gln from being used to generate hexosamine (through glutamine-fructose-6-phosphate transaminase), purines (amidophosphorybosyl transferase or formylglycinamide ribonucleotide amidotransferase), pyrimidine and Glu^13,14^. DON was tested in patients with cancer; however, those studies failed because of the poor pharmacological properties and gastrointestinal toxicities^15^ of the drug. To overcome these limitations, recent efforts led to the creation of sirpiglenastat (DRP-104), a pro-drug version of DON that circumvents the toxicities of DON while retaining the covalent and irreversible properties of DON^16–19^. Early-phase clinical trials are examining the efficacy of DRP-104 as a single agent or in combination with immune checkpoint inhibitors for non-small cell lung cancer and squamous cell carcinoma of the head and neck (ClinicalTrials.gov registration: NCT04471415)^20^.

In this study, we demonstrated that both DON and DRP-104 caused a metabolic crisis in PDAC by globally impairing Gln metabolism, resulting in a significant decrease in proliferation. In addition, we observed a significant reduction in tumor growth in several in vivo PDAC models using DRP-104 as a monotherapy. Analysis of DRP-104-treated tumors demonstrated an increase in extracellular signal-regulated kinase (ERK) signaling. Mechanistically, we found that ERK signaling was increased as a compensatory mechanism through the upregulation of several receptor tyrosine kinase (RTK) receptors, such as Axl and members of the ErbB family. Combinatorial treatment with DRP-104 and trametinib (an MAP kinase (MAPK)/ERK kinase 1 and 2 inhibitor) led to an increase in survival in a syngeneic PDAC model. These preclinical results suggest that broadly targeting Gln metabolism could provide an alternative therapeutic avenue for PDAC.

## Results

### DON creates a metabolic crisis in pancreatic cancer

**Fig. 1:**
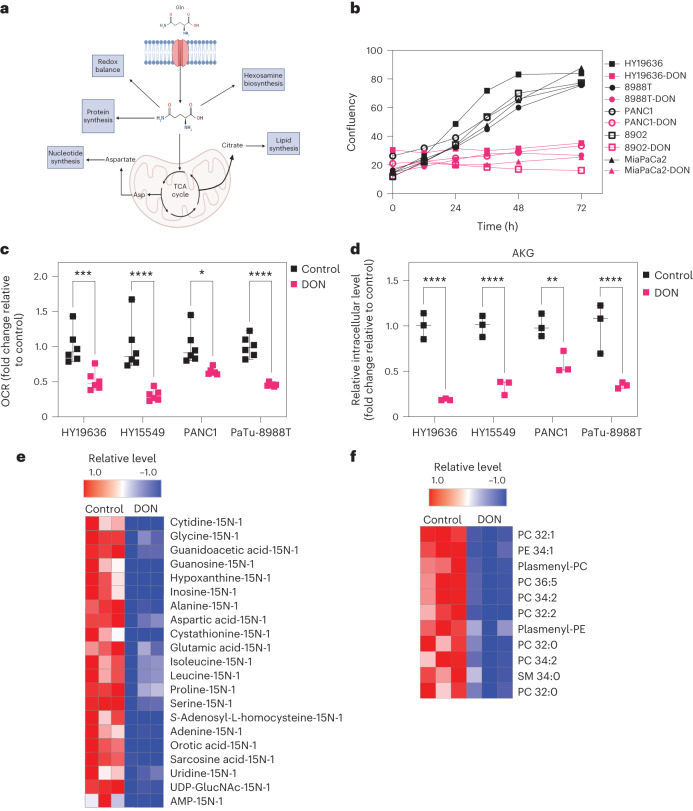
DON creates a metabolic crisis in pancreatic cancer. **a**, As Gln has pleiotropic roles in cellular metabolism, we hypothesized that using a broad-acting Gln antagonist, such as DON or DRP-104, could prevent the rapid metabolic adaptation seen in these tumors. Diagram created with Biorender.com. **b**, Panel of human and murine PDAC cell lines treated with DON (25 µM) for 72 h. Data are displayed as confluency. Data are displayed as the mean of *n* = 3 biological replicates per condition per cell line. **c**, Basal OCR was decreased in mouse (HY19636, HY15549) and human (PANC1, PaTu-8988T) PDAC cells on DON treatment. HY19636, ***P = 0.0003; HY15549, ****P < 0.0001; PANC1, *P = 0.0106; PaTu-8988T, ****P < 0.0001. Statistics were derived from *n* = 6 samples per condition from two independent experiments (three samples per experiment). Data are shown as the mean ± s.e.m. of *n* = 6 biological replicates per condition. Significance was determined using a two-way analysis of variance (ANOVA) using a Holm-Šídák’s multiple comparisons test. *P = 0.0106, ****P < 0.0001. **d**, Total pool size of intracellular AKG in PDAC cells in the presence or absence of DON (25 µM) in HY19636, HY15549, PANC1 and PaTu-8988T cells. Data were obtained using gas chromatography–mass spectrometry (GC–MS) and normalized to an internal standard (norvaline) and cell number. Statistics were derived from a representative experiment with *n* = 3 independent samples per condition. The GC–MS experiment was performed twice with similar results. Data are shown as the mean ± s.e.m. of *n* = 3 replicates. Significance was determined using a two-way ANOVA with a Holm-Šídák’s multiple comparisons test. **P = 0.0071, ****P < 0.0001. **e**, Isotopolog abundance in cells labeled with ^15^N-Gln after DON treatment in HY19636 cells. All the metabolites in this heatmap have a statistical significance of at least *P* < 0.05. Significance was determined using the Wald test (DESeq2) corrected for multiple comparisons. The columns are biological replicates in triplicate and the rows represent metabolites. Red indicates the maximum level (1.0) and blue is lower (−1.0) than the mean for each metabolite between control and DON treatment. **f**, Relative lipid levels in cells treated with DON after 24 h. All the lipids presented in this heatmap have a statistical significance of at least *P* < 0.005. Significance was determined using the Wald test corrected for multiple comparisons. Columns are biological replicates in triplicate; rows represent metabolites. Red indicates the maximum level (1.0); blue is lower (−1.0) relative to the mean for each metabolite between control and DON treatment. For the complete list of lipid species and full annotations, refer to the source data. Heatmaps generated with Morpheus. Source data

**Extended Data Fig. 1:**
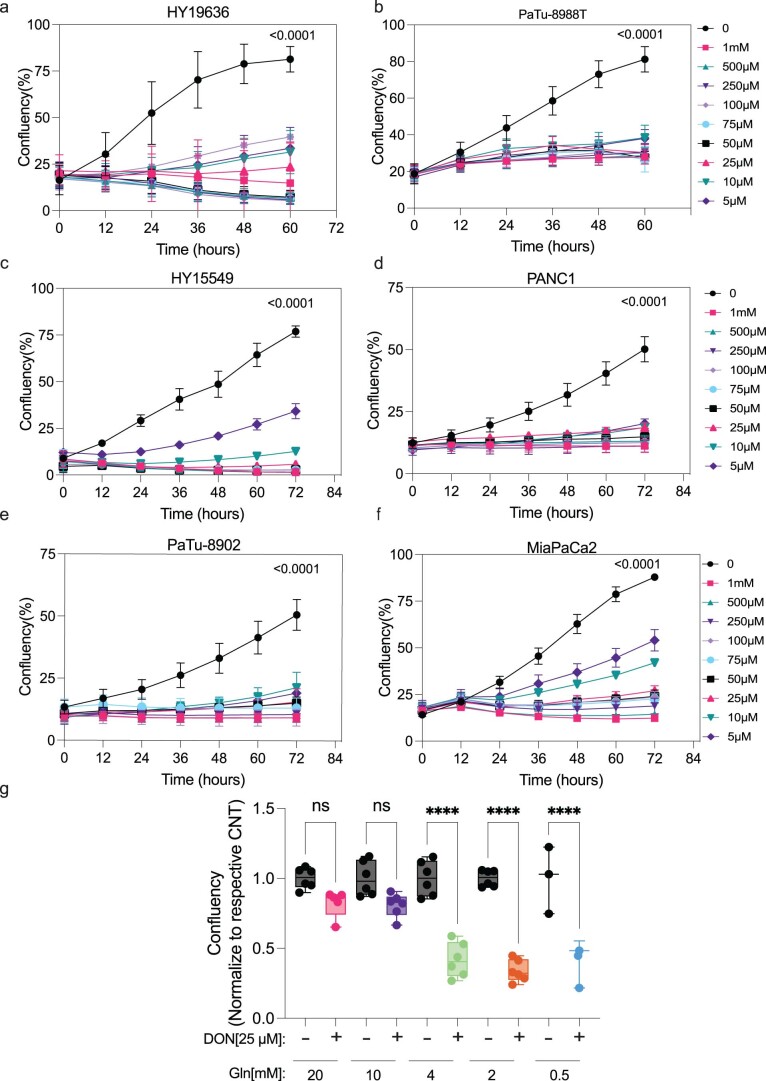
DON inhibits cellular proliferation in a panel of PDAC lines. Cell growth of human and murine pancreatic cancer cells (**a**) HY19636, (**b**) PaTu-8988T, (**c**)HY15549, (**d**)PANC1, (**e**)PaTu-8902 and (**f**)MiaPaCa2 exposed to DON for 72 hours at various concentrations ([DON, µM]: 500, 250, 100, 75, 50, 25, 10, and 5). Data plotted as confluency (geometric mean and error, n = 3). Significance was determined by two-way ANOVA using Dunnett’s multiple comparisons test. All the p values are included in the data source. (**g**) HY19636 cells were treated with DON (25 µM) for 7 days at various concentrations of glutamine (20 mM, n = 5; 10 mM, n = 6; 4 mM, n = 6; 2 mM, n = 6; 0.5 mM, n = 3). Representation of one representative experiment. Every condition was normalized to the control of each respective glutamine concentration. Data is displayed as mean ± s.e.m. The box plots extend from the 25th to 75th percentiles. The whiskers represent the smallest to largest values. The line in the middle of the box is plotted at the median. Significance was determined by ordinary one-way ANOVA using Tukey’s multiple comparisons test. P value: ****: <0.0001. All the p values are included in the data source. Source data

**Extended Data Fig. 2:**
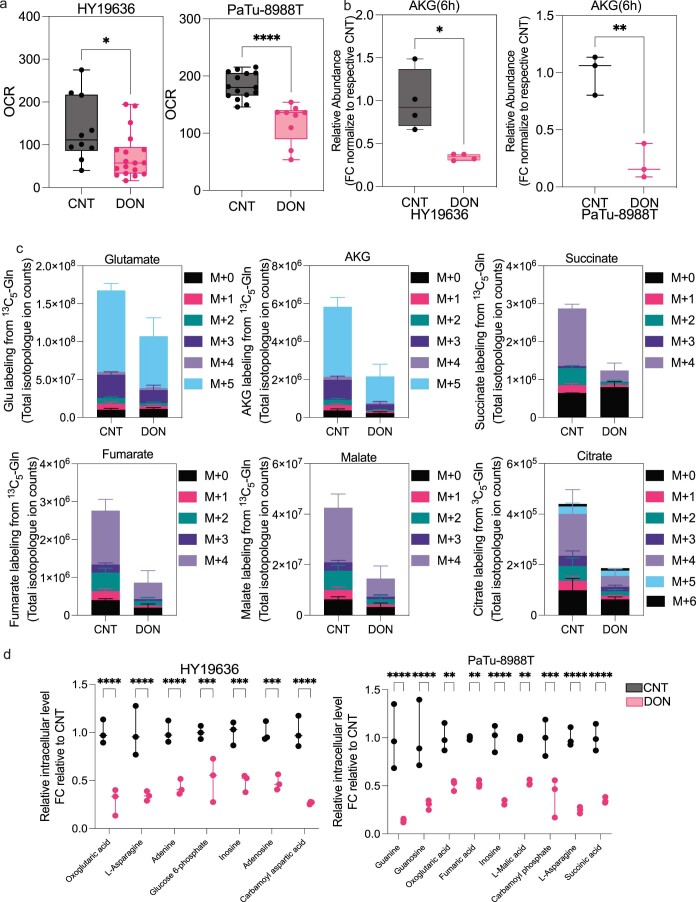
DON rapidly reduces TCA cycle metabolism in PDAC. (**a**) Basal oxygen consumption rate is decreased in HY19636 and PaTu-8988T after 6 hours of DON treatment (25 µM). For HY19636, VEH, n = 10; DON, n = 18; P Value: **: 0.0056. For PaTu-8988T, VEH, n = 15; DRP-104, n = 9. P value: ****:0.0001 Significance was determined using unpaired t-test (two-tailed). The box plots extend from the 25th to 75th percentiles. The whiskers represent the smallest to largest values. The line in the middle of the box is plotted at the median. (**b**) Total ion counts of alpha-ketoglutarate (AKG) in HY19636 and PaTu-8988T after 6 hours treatment of DON (25 µM). Data relative to control and internal standard (norvaline). For HY19636, VEH, n = 4; DON, n = 4; P value: 0.0104 (*). Data displayed as mean ± s.e.m of n = 4. For PaTu-8988T, VEH, n = 3; DON, n = 3; P value: 0.0041 (**). Data displayed as mean ± s.e.m of n = 3. Significance was determined by unpaired t-test (two-tailed). The box plots extend from the 25th to 75th percentiles. The whiskers represent the smallest to largest values. The line in the middle of the box is plotted at the median. (**c**) Total ion counts of isotopologues of U-^13^C-Glutamine labeling of the indicated metabolites in HY19636 cells. VEH, n = 3; DRP-104, n = 3. Data displayed as mean ± s.e.m. Significance was determined by one-way ANOVA using Tukey’s multiple comparisons test. All the statistical test and values are included in the data source. (**d**) Relative abundance of metabolites in central carbon metabolism, TCA cycle, and nucleotide metabolism after DON treatment in HY19636 and PaTu-8988T. Data was obtained through LCMS and represented as fold change relative to control. VEH, n = 3; DRP-104, n = 3. Data displayed as mean ± s.e.m. Significance was determined by ordinary two-way ANOVA using Šídák’s multiple comparisons test multiple comparisons test. All the statistical test and values are included in the data source. All the statistical test and values are included in the data source. Source data

We hypothesized that the broad antagonism of Gln use with DON might increase efficacy in PDAC compared to previous efforts to target Gln metabolism in PDAC tumors (Fig. 1a). To test this hypothesis, we first examined the role of DON using a panel of PDAC lines derived from human or mouse (KPC-LSL*Kras*^G12D/+^; *Trp53*^L/+^; *P48*^Cre^). We first demonstrated that DON treatment led to a significant decrease in cellular proliferation across several PDAC tumors (Fig. 1b and Extended Data Fig. 1a–h). This reduction in proliferation was mitigated at higher doses of Gln, which is consistent with previous reports (Extended Data Fig. 1g)^19^. To investigate if this decrease in cellular proliferation was due to a disruption of metabolic homeostasis, we assessed mitochondrial function by measuring the oxygen consumption rate (OCR) in several PDAC lines in the presence or absence of DON. Strikingly, DON treatment significantly reduced OCR levels, indicating decreased oxidative phosphorylation (Fig. 1c). Indeed, we observed that this decrease in OCR was achieved within a short incubation time, suggesting that it is independent of proliferation (Extended Data Fig. 2a,b). Tracing studies using U-^13^C_5_-Gln demonstrated a significant decrease in both the labeled fraction and total pool sizes of TCA cycle intermediates. The reduction in the M3 fraction of AKG and Glu suggested that the TCA cycle is defective (Extended Data Fig. 2c). More importantly, these data indicated that DON-treated cells are unable to effectively compensate for the suppression of mitochondrial metabolism with other fuel sources, such as glucose as observed in the M0 fraction of Glu and other TCA intermediates (Extended Data Fig. 2c). Furthermore, we observed a reduction in the total levels of AKG and other Gln-related metabolites in several PDAC cell lines (Fig. 1d, Extended Data Fig. 2c–e and Extended Data Fig. 4a–d).

**Extended Data Fig. 3:**
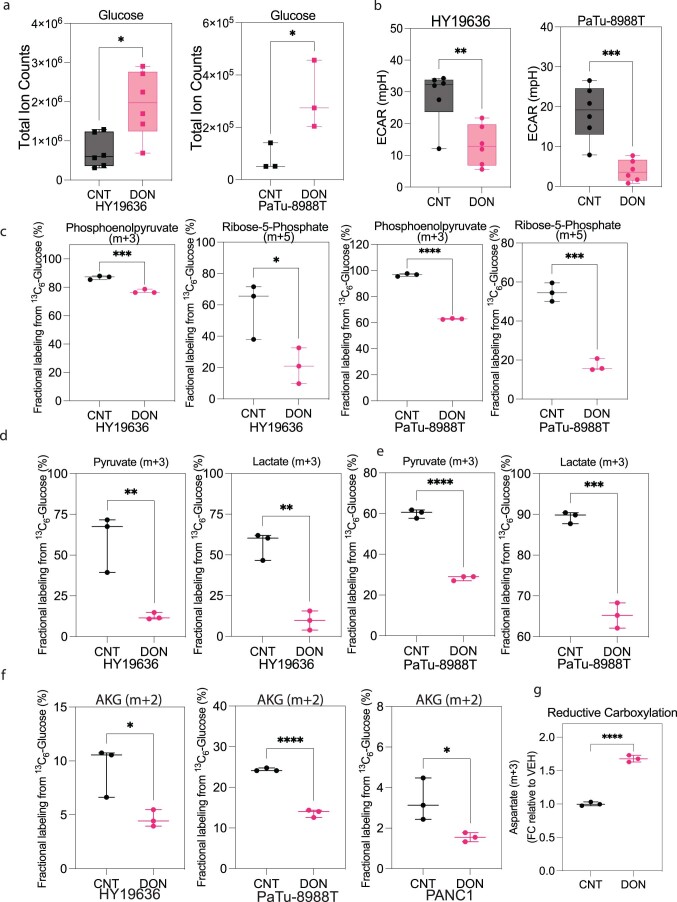
DON impairs glucose metabolism in PDAC. (**a**) Total ion counts of intracellular D-Glucose in PDAC cells in the presence or absence of DON (25 µM, 24 hours) (HY19636 and PaTu-8988T). For HY19636, VEH, n = 6; DRP-104, n = 6; P value: 0.0101 (*). For PaTu-8988T, VEH, n = 3; DRP-104, n = 3; P value: 0.0463 (*). Data was obtained through LC-MS and normalized to cell number. Data displayed as mean ± s.e.m. and significance were determined was unpaired t-test two-tailed. (**b**) Extracellular acidification rate (ECAR) is decreased in HY19636 and PaTu-8988T after 24 hours of treatment of DON (25 µM). For HY19636, VEH, n = 6; DRP-104, n = 6; P value: 0.005 (**). For PaTu-8988T, VEH, n = 6; DRP-104, n = 6; P value: 0.0006 (**). Significance was determined by unpaired t-test (two-tailed). (**c**) Fractional labeling from U-^13^C_6_-Glucose of phosphoenolpyruvate (m + 3) and ribose-5-phosphate (m + 5) after DON treatment (25 µM) in HY19636 and PaTu-8988T. For HY19636, VEH, n = 3; DRP-104, n = 3; P values: 0.0009 (***); 0.0384 (*). For PaTu-8988T, VEH, n = 3; DRP-104, n = 3; P values: <0.0001 (****); 0.0003 (***). Data was obtained through LCMS and plotted as percentage. Data displayed as mean ± s.e.m and significance were determined was unpaired t-test (two-tailed). The box plots extend from the 25th to 75th percentiles. The whiskers represent the smallest to largest values. The line in the middle of the box is plotted at the median. (**d**) Contribution of U-^13^C_6_-Glucose to pyruvate and lactate after DON (25 µM, 24 hours) treatment in HY19636, VEH, n = 3; DRP-104, n = 3; P value:0.01 (**), 0.0014 (**) and (**e**) PaTu-8988T, VEH, n = 3; DRP-104, n = 3; P value: <0.0001 (****), 0.003 (***). Data was obtained through LCMS and plotted as a percentage. Data displayed as mean ± s.e.m. and significance were determined was unpaired t-test (two-tailed). (**f**) Fractional labeling of αKG in cells labeled with [U-^13^C_6_]-Glucose of cells after DON treatment in HY19636, PaTu-8988T and PANC1 cells. For all cell lines, VEH, n = 3, DRP-104, n = 3. P values: 0.0296(*); < 0.0001 (****); 0.043 (*) Data was obtained through LCMS and plotted as a percentage. Data displayed as mean ± s.e.m. and significance were determined was unpaired t-test (two-tailed). (**g**) Relative levels of Aspartate (m + 3) derived from [U-^13^C_5_]-Glutamine after DON treatment in PaTu-8988T, suggesting that TCA is been reductive instead of oxidative as observed by the odd numbers of labeled carbons. Data was collected through GC-MS, the percentage of incorporation was normalized to control. Data displayed as mean ± s.e.m. and significance were determined was unpaired t-test (two-tailed). Source data

Interestingly, we also observed an increase in intracellular d-glucose levels in DON-treated cells (Extended Data Fig. 3a). However, this increase in glucose level did not result in higher glycolytic activity; instead, there was a significant decrease in glycolysis, as measured by extracellular acidification rate or decreased incorporation of labeled glucose into glycolytic intermediates, pyruvate, lactate or TCA cycle intermediates (Extended Data Fig. 3b–f). Lastly, DON-treated cells also exhibited increased levels of reductive carboxylation, a process well-documented to maintain citrate levels in the context of hypoxia or mitochondrial impairment, further supporting that these cells were experiencing mitochondrial dysfunction (Extended Data Fig. 3g)^21–24^. Together, these data suggest that the profound effects of DON on central carbon metabolism are far broader than just inhibiting GLS.

One of the significant differences between DON and GLS inhibitors is that DON should impair the contribution of Gln amino-nitrogens and amido-nitrogens in addition to its carbon skeleton. To test if DON could prevent nitrogen use, we cultured PDAC cells with amino-^15^N-Gln in the presence or absence of DON. Like the inhibition of carbon use, DON significantly reduced the incorporation of amino-^15^N-Gln into downstream metabolites, including those associated with nonessential amino acid, hexosamine and nucleotide metabolism (Fig. 1e). This was consistent with the reduction in nucleotide pools we observed. Notably, DON treatment significantly reduced the levels of citrate, which resides at the nexus of the TCA cycle and lipid metabolism (Extended Data Fig. 5a–c). Therefore, we performed lipidomics and observed that DON treatment led to significant decreases in membrane-enriched lipids, such as phosphatidylcholines, phosphatidylethanolamines and sphingomyelin (Fig. 1f and Extended Data Fig. 5d,e).

To understand the stress response(s) that result from the DON-mediated metabolic crisis, we performed bulk RNA sequencing (RNA-seq) and observed a significant induction of several members of the integrated stress response pathway, such as Gadd45a, Ddit3, the Atf family and Ppp1r15a^25^ (Extended Data Fig. 6a,b). Consistent with the transcriptomic data, we observed robust ATF4 activation and phosphorylation of eIF2α, suggesting a decrease in global translation (Extended Data Fig. 6c). Consistent with a decrease in protein synthesis, the contribution of Gln to proteinogenic amino acids (for example, Glu, aspartate (Asp), proline) is significantly decreased with DON treatment (Extended Data Fig. 6d). Additionally, we saw a reduction in the total levels of uridine 5′-diphospho (UDP) *N*-acetylglucosamine and protein *O*-GlcNAcylation, which is consistent with impairment of the hexosamine biosynthetic pathway (Extended Data Fig. 6e,f). Together, these results show that DON treatment elicits a metabolic crisis in PDAC cell lines by inhibiting the activity of multiple metabolic pathways important for cell proliferation.

### DRP-104 reduces tumor growth in several PDAC models

**Fig. 2:**
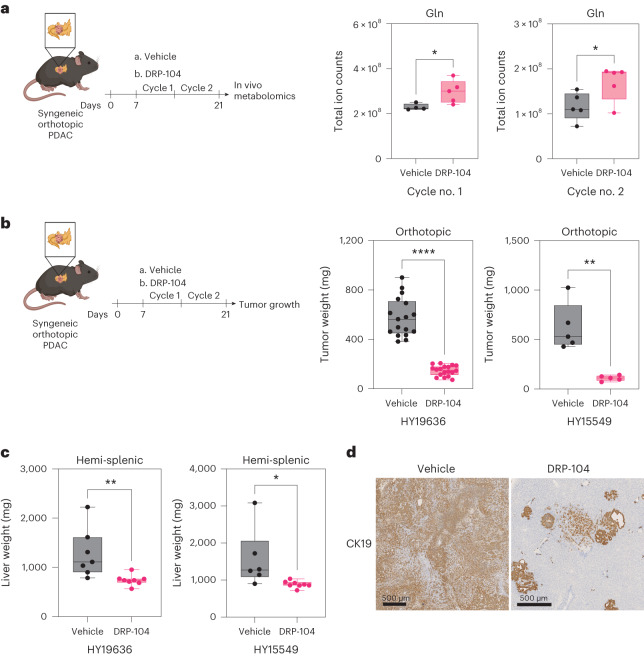
DRP-104 reduces tumor growth and liver colonization in syngeneic PDAC models. **a**, In vivo measurement of Gln through liquid chromatography (LC)–MS in a DRP-104-treated syngeneic PDAC model. Data are presented as total ion counts and normalized to tumor weight and internal standards. Cycle no. 1: vehicle, *n* = 4 tumors from biologically independent mice; DRP-104, *n* = 5 tumors from biologically independent mice; *P* = 0.0370. Cycle no. 2: vehicle, *n* = 5; DRP-104, *n* = 5; *P* = 0.0483. Significance was determined using a two-tailed unpaired *t*-test. The box plots extend from the 25th to the 75th percentile. The whiskers represent the smallest to largest values. The line in the middle of the box represents the median. **b**, KPC-derived (HY19636 and HY15549) cells were injected into the pancreata of B6J mice. Mice were treated daily either with vehicle or DRP-104 (3 mg kg^−1^) intraperitoneally for two cycles (5 days on, 2 days off). Samples were collected 21 days after transplantation for downstream evaluations. For HY19636 cells, data were pooled from two independent experiments (vehicle, *n* = 17 tumors from biologically independent mice; DRP-104, *n* = 18 tumors from biologically independent mice; ****P < 0.0001. For HY15549: vehicle, *n* = 5 tumors from biologically independent mice; DRP-104, *n* = 5 tumors from biologically independent mice; **P = 0.0015. Significance was determined using a two-tailed unpaired *t*-test. The box plots extend from the 25th to the 75th percentile. The whiskers represent the smallest to largest values. The line in the middle of the box represents the median. **c**, Weight of the livers after treatment with vehicle or DRP-104 (3 m kg^−1^) in B6J mice. DRP-104 treatment started 3 days after the hemi-splenectomy. For HY19636 cells: vehicle, *n* = 7 from biologically independent mice; DRP-104, *n* = 8 from biologically independent mice; **P = 0.009. For HY15549 cells: vehicle, *n* = 6 from biologically independent mice; DRP-104, *n* = 8 from biologically independent mice; *P = 0.0314. Significance was determined using a two-tailed unpaired *t*-test. The box plots extend from the 25th to the 75th percentile. The whiskers represent the smallest to largest values. The line in the middle of the box represents the median. **d**, Representative images of liver metastasis (from biologically independent mice) visualized using CK19 staining. CK19 staining was performed in all the samples listed in Fig. 2c and showed similar results. Source data

The metabolic effects of DON and the decrease in cell proliferation led us to hypothesize that this approach would be effective in several in vivo models. However, previous preclinical and clinical studies showed poor pharmacological properties of DON and toxicity issues with regard to the gastrointestinal tract, leading to these approaches being abandoned in patients. To overcome these challenges, we used a pro-drug version of DON, DRP-104, to treat several PDAC mouse models. We first validated that DRP-104 caused a metabolic crisis in vitro similar to DON, impacting multiple critical metabolic pathways (Extended Data Fig. 7a). Most importantly, DRP-104 treatment led to an accumulation of tumor Gln levels in vivo (Fig. 2a), supporting its on-target activity as an inhibitor of Gln use. Remarkably, DRP-104 significantly reduced tumor growth in two independent orthotopic syngeneic PDAC models after two cycles of treatment (Fig. 2b). To evaluate the efficacy of DRP-104 beyond the primary tumor, we used a hemi-splenectomy model to assess liver metastasis in a syngeneic model. DRP-104 treatment significantly reduced liver colonization in two independent models (Fig. 2c,d). These data indicate that DRP-104 effectively delays tumor growth and prevents metastatic colonization in PDAC.

**Fig. 3:**
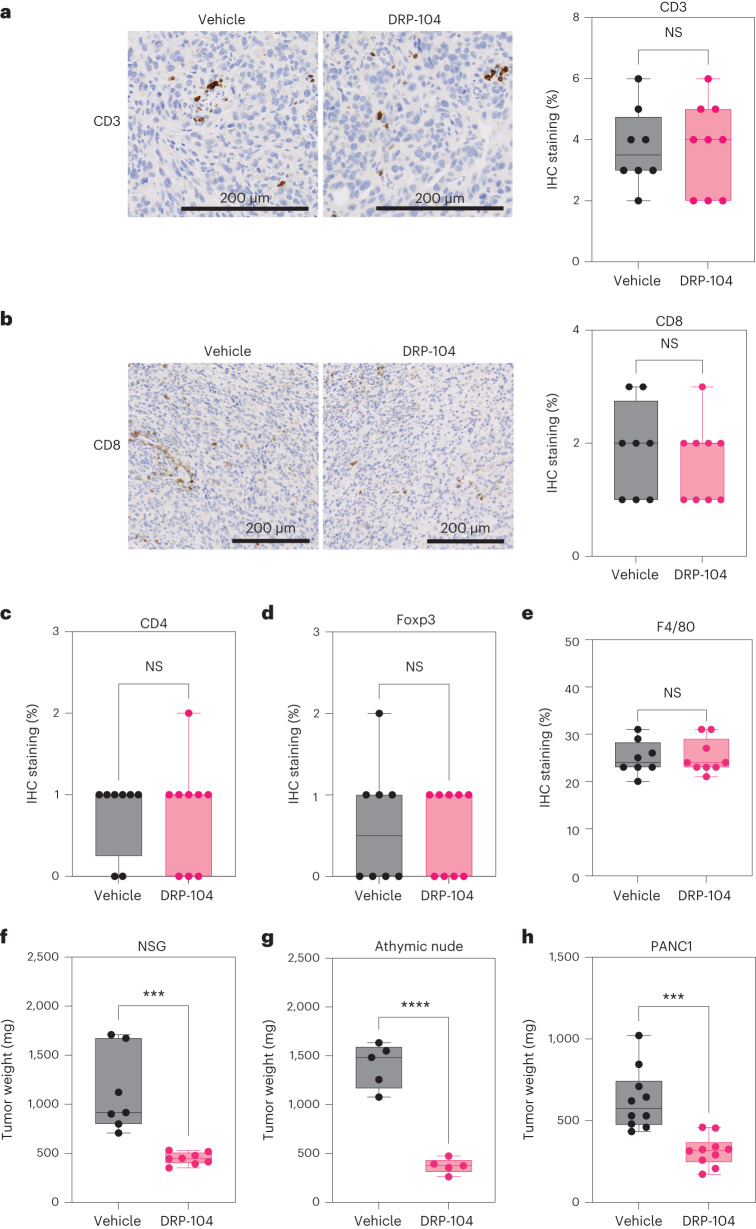
The adaptive immune system is not required for DRP-104 antitumor properties in PDAC. **a**,**b**, Representative images and quantification of CD3 (**a**) and CD8 (**b**) staining in DRP-104-treated and control tumors (HY19636) (vehicle, *n* = 8 tumors from biologically independent mice; DRP-104, *n* = 9 tumors from biologically independent mice). A two-tailed unpaired *t*-test showed no significance. CD3: *P* = 0.96; CD8: *P* = 0.58. IHC, immunohistochemistry. **c**, Quantification of CD4 levels in DRP-104-treated tumors (HY19636) (vehicle, *n* = 8 tumors from biologically independent mice; DRP-104, *n* = 9 tumors from biologically independent mice). A two-tailed unpaired *t*-test showed no significance (NS) (*P* = 0.92). **d**, Quantification of Foxp3 levels in DRP-104-treated tumors (HY19636) (vehicle, *n* = 8 tumors from biologically independent mice; DRP-104, *n* = 9 tumors from biologically independent mice). A two-tailed unpaired *t*-test showed no significance (*P* = 0.8257). **e**, Quantification of F4/80 levels in DRP-104-treated tumors (HY19636) (vehicle, *n* = 8 tumors from biologically independent mice; DRP-104, *n* = 9 tumors from biologically independent mice). A two-tailed unpaired *t*-test showed no significance (*P* = 0.90). **f**, KPC-derived (HY19636) cells were injected into the pancreata of NSG mice. Vehicle, *n* = 5 tumors from biologically independent mice; DRP-104, *n* = 5 tumors from biologically independent mice (***P = 0.0005). Significance was determined using a two-tailed unpaired *t*-test. **g**, KPC-derived (HY19636) cells were injected into the pancreata of athymic nude mice. Mice were treated daily either with vehicle or DRP-104 (3 mg kg^−1^) intraperitoneally for two cycles (5 days on, 2 days off). Samples were collected 21 days after transplantation for downstream evaluation. Vehicle, *n* = 5 tumors from biologically independent mice; DRP-104, *n* = 5 tumors from biologically independent mice. ****P < 0.0001. Significance was determined using a two-tailed unpaired *t*-test. **h**, PANC1 cells were injected into the pancreata of athymic nude mice. Treatment started 14 days after implantation. Samples were collected 21 days after transplantation for downstream evaluation. Vehicle, *n* = 10 tumors from biologically independent mice; DRP-104, *n* = 10 tumors from biologically independent mice. ***P = 0.0002. Significance was determined using a two-tailed unpaired *t*-test. In Fig. 3a–h, the box plots extend from the 25th to the 75th percentile. The whiskers represent the smallest to largest values. The line in the middle of the box represents the median. Source data

**Fig. 4:**
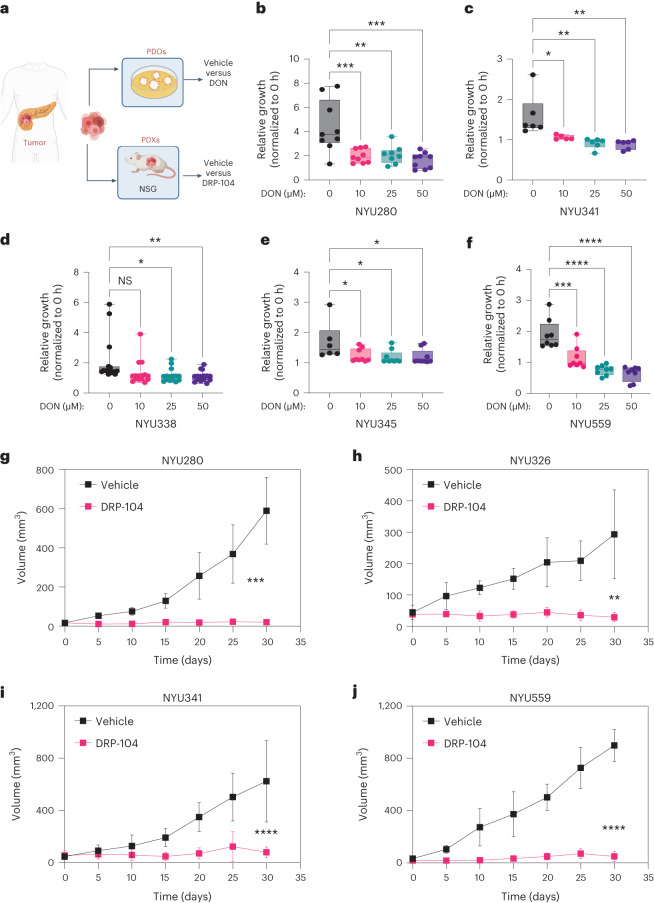
DON/DRP-104 diminishes proliferation in PDAC organoids and PDX models. **a**, PDAC organoids and PDX models were generated from human pancreatic cancer samples. PDAC organoids were treated with DON at the indicated doses. **b**, NYU280 organoids were treated with increasing concentrations of DON (10 µM, 25 µM and 50 µM) for 7 days. Data were obtained from three independent experiments. ***P < 0.0007 (control versus 10 µM of DON); **P = 0.0016 (control versus 25 µM of DON); ***P < 0.0001 (control versus 50 µM of DON). **c**, NYU341 organoids were treated with increasing concentrations of DON (10 µM, 25 µM and 50 µM) for 7 days. Data were obtained from three independent experiments. *P = 0.0172 (control versus 10µM of DON); **P = 0.0017 (control versus 25 µM of DON); **P = 0.0011 (control versus 50 µM of DON). **d**, NYU338 organoids were treated with increasing concentrations of DON (10 µM, 25 µM and 50 µM) for 7 days. Data were obtained from three independent experiments. *P* = 0.0513 (not significant (NS)); *P = 0.0133; **P = 0.0081. **e**, NYU345 organoids were treated with increasing concentrations of DON (10 µM, 25 µM and 50 µM) for 7 days. Data were obtained from three independent experiments. *P = 0.036 (control versus 10 µM of DON); *P = 0.0202 (control versus 25 µM of DON); *P = 0.0214 (control versus 50 µM of DON). **f**, NYU559 organoids were treated with increasing concentrations of DON (10 µM, 25 µM and 50 µM) for 7 days. Data were obtained from three independent experiments. ***P = 0.0003 (control versus 10 µM of DON); ****P= 0.0001 (control versus 25µM of DON); ****P = 0.0001 (control versus 50 µM of DON). For all PDO studies, growth was measured as confluency over time and normalized to *t* = 0 h. Significance for Fig. 4a–f was determined using a one-way ANOVA with a Dunnett’s multiple comparisons test from at least *n* = 6 independent biological samples. PDX species were implanted subcutaneously in the flank of NSG mice. Mice were treated daily either with vehicle or DRP-104 (3 mg kg^−1^) intraperitoneally for four cycles (5 days on, 2 days off). **g**, NYU280: vehicle, *n* = 4; DRP-104, *n* = 5; ***P < 0.0001. **h**, NYU326: vehicle, *n* = 5; DRP-104, *n* = 4; **P = 0.008. **i**, NYU341: vehicle, *n* = 11; DRP-104, *n* = 11; ****P < 0.0001. **j**, NYU559: vehicle, *n* = 4; DRP-104, *n* = 4; ****P < 0.0001. For all PDX studies, tumors were measured every 5 days. In Fig. 4g–j, data were plotted as the geometric mean, s.e.m. and s.d. In Fig. 4g–j, all samples were tumors from biologically independent mice and significance was determined using a two-tailed unpaired *t*-test. Source data

Next, we set out to evaluate if the antitumor properties of DRP-104 were influenced by extrinsic factors in the TME. We evaluated the presence of cancer-associated fibroblasts (CAFs) and subtypes associated with PDAC using subsets defined by podoplanin, aSMA and platelet-derived growth factor receptor (PDGFR), and found no significant changes in DRP-104-treated tumors (Extended Data Fig. 8a–d). To dissect the immune contribution to the DRP-104 effect, we measured the infiltration of immune populations in DRP-104-treated tumors. Consistent with previous studies, PDAC tumors had basally low levels of T cell infiltration. DRP-104 did not increase the levels of CD3, CD8, CD4, Foxp3 or macrophages in the tumors (Fig. 3a–e). To functionally assess whether the antitumor response required a functional adaptive immune system, we examined DRP-104 in immunodeficient models (NOD-*scid* gamma (NSG) and athymic nude models). Unlike many other tumor types that showed the important contribution of the adaptive immune system^17,19,26^, the antitumor properties of DRP-104 in PDAC were preserved in both the athymic nude and NSG models (Fig. 3f,g). Similar results were obtained using a human-derived line (PANC1) in the athymic nude mouse model (Fig. 3h). To better understand the impact of Gln antagonism in other clinically relevant models, we tested Gln antagonism in a set of patient-derived organoids (PDOs) and patient-derived xenografts (PDXs) (Fig. 4a). DON demonstrated a reduction in proliferation in a panel of PDAC PDOs (Fig. 4b–f). Consistent with the PDO data and the other models tested (syngeneic, immune-deficient), we observed a significant decrease in tumor growth in multiple PDX models (Fig. 4g–j). Taken together, these data suggest that DON/DRP-104 is broadly effective in decreasing PDAC growth across multiple models.

### MAPK signaling is activated by DRP-104 treatment as a resistance mechanism

**Fig. 5:**
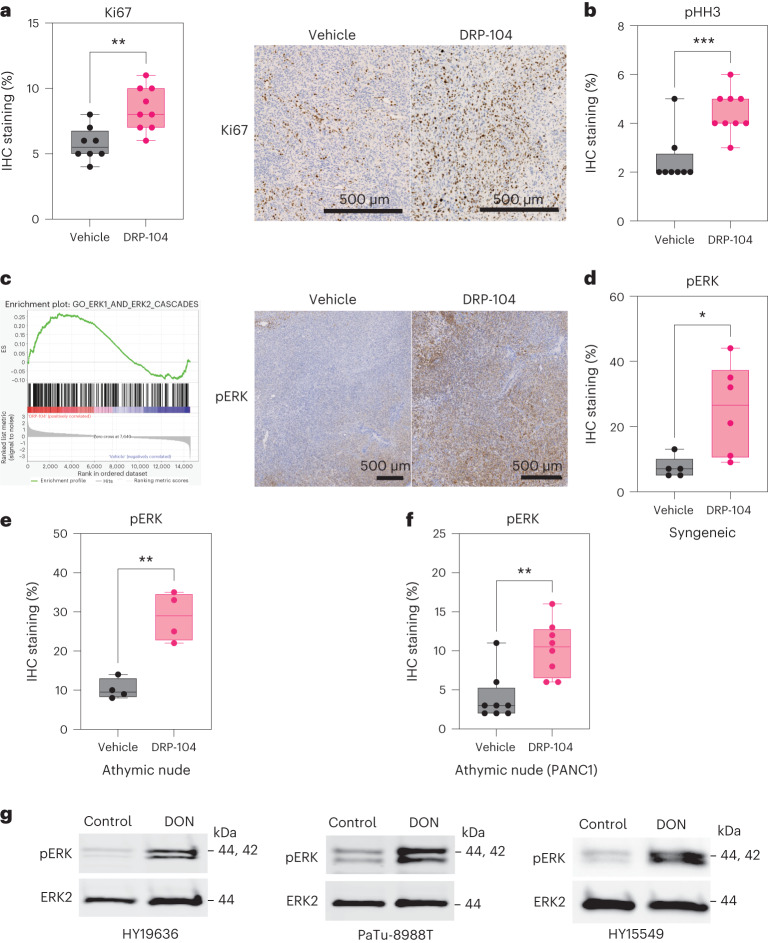
MAPK signaling is activated by DRP-104 treatment as a resistance mechanism. **a**, Quantification of Ki67 levels in DRP-104-treated tumors (HY19636). Vehicle, *n* = 8 tumors from biologically independent mice; DRP-104, *n* = 9 tumors from biologically independent mice; **P = 0.0021. **b**, Quantification of phospho-histone H3 (pHH3) levels in DRP-104-treated tumors (HY19636). Vehicle, *n* = 8 tumors from biologically independent mice; DRP-104, *n* = 9 tumors from biologically independent mice; ***P = 0.0009. Significance was determined using a two-tailed unpaired *t*-test. **c**, GSEA of transcripts between DRP-104-treated and control tumors demonstrated enrichment in the ERK1 and ERK2 cascades (GO:0070371). Enrichment score (ES) = 0.2; normalized ES = 1.25, false discovery rate *q* = 0.05. IHC analysis of phospho-ERK (pERK) in a representative PDAC tumor. For the bulk RNA-seq studies, three randomly selected tumors were used per group. **d**, Quantification of pERK in the syngeneic model with HY19636 cells. Vehicle, *n* = 5 tumors from biologically independent mice; DRP-104, *n* = 6 tumors from biologically independent mice; *P = 0.0214. Significance was determined using a two-tailed unpaired *t*-test. **e**, pERK levels in athymic nude mice with HY19636 cells. Vehicle, *n* = 4 tumors from biologically independent mice; DRP-104, *n* = 4 tumors from biologically independent mice; **P = 0.0016. Significance was determined using a two-tailed unpaired *t*-test. **f**, pERK levels in athymic nude mice with PANC1 cells. Vehicle, *n* = 8 tumors from biologically independent mice; DRP-104, *n* = 8 tumors from biologically independent mice; **P = 0.002. Significance was determined using a two-tailed unpaired *t*-test. **g**, Immunoblot of pERK and ERK2 (loading control) in HY19636, HY15549 and PaTu-8988T cells treated with DON (25 µM) for 24 h. Representative immunoblot of two independent experiments. Source data

While there was substantial single-agent activity using DRP-104, residual tumors at the experimental end point displayed higher levels of proliferation markers compared to vehicle, suggesting that these tumors developed therapeutic resistance (Fig. 5a,b). We aimed to identify potential mechanisms of adaptations to the broad Gln perturbation. First, we evaluated the levels and activity of Gln synthetase (GS) in vitro, the key enzyme responsible for de novo Gln synthesis, which has been identified as an essential factor for adaptation in PDAC during nutrient starvation^11,27^. We observed a heterogeneous expression of GS in both human and mouse PDAC lines, with most not displaying increased expression on DON treatment. To functionally elucidate GS activity we assessed the de novo synthesis of Gln using tracing studies. GS expression did not demonstrate an increase in unlabeled Gln, suggesting that this is not an effective compensatory resistance mechanism in this context (Extended Data Fig. 9a–c). We also evaluated GS levels in vivo and found no difference in GS expression on DRP-104 treatment in syngeneic tumors at the end point (Extended Data Fig. 9d). Together, these data suggest that GS is not an effective compensatory resistance mechanism in this context.

We set out to gain a deeper understanding of how cells adapt to the perturbation of Gln metabolism, with a particular focus on how they obtain sufficient nitrogen. Previously, we observed that in the context of alanine deficiency, PDAC cells rely on Gln to support metabolism (including as a key nitrogen source)^28^. We sought to investigate if the converse was true in the context of Gln perturbation. To achieve that goal, we labeled cells with ^15^N-alanine and found that DON-treated cells increased alanine incorporation into the nitrogen of key TCA cycle intermediates such as Glu (Extended Data Fig. 9d). This observation suggests that other carbon and nitrogen sources, like alanine, could potentially compensate to mitigate the metabolic impacts of DON.

To dissect the potential mechanisms of resistance of DRP-104 in vivo, we evaluated the transcriptional profile of DRP-104-treated tumors at the end point. These data revealed an enrichment in MAPK (ERK) signaling, response to reactive oxygen species (ROS), epithelial–mesenchymal transition (EMT) and oxidative phosphorylation on DRP-104 treatment from gene set enrichment analysis (GSEA) (Fig. 5c and Extended Data Fig. 10a). Given the well-established role of ERK signaling in cell survival and metabolism, we hypothesized that ERK signaling might act as a compensatory mechanism on Gln antagonism^29,30^. Indeed, activation of this pathway supports central carbon metabolism in PDAC^33,31^. Immunohistochemical analysis confirmed induction of pERK on DRP-104 treatment in both the syngeneic and athymic mouse models (Fig. 5d–f). Furthermore, we observed the induction of pERK in multiple PDAC lines on DON exposure in cell culture, suggesting that this is a cell-intrinsic mechanism (Fig. 5g).

To further examine and dissect the mechanisms that drive ERK activation on DON/DRP-104 treatment, we characterized potential activators of ERK. First, as high levels of ROS can lead to ERK activation through the decreased expression of the negative regulator dual specificity phosphatase 6 (DUSP6) (ref. ^32^), we confirmed that DON treatment resulted in increased ROS levels, decreased reduced glutathione levels and decreased DUSP6 expression (Extended Data Fig. 10b–f). To verify if ROS are a major driver of ERK activation on DON treatment, we supplemented DON-treated cells with the antioxidant *N*-acetyl cysteine (NAC). Supplementation with NAC did not prevent ERK activation on DON treatment, suggesting that other drivers lead to ERK pathway induction (Extended Data Fig. 10f,g). To assess for possible upstream activators, we performed RTK activation arrays in PDAC cells treated with DON. Oncogenic RTKs have been linked to metabolic plasticity and therapy response in cancer^33–36^. Indeed we found that phosphorylation of various RTKs, such as Axl and members of the ErbB family, was increased after DON treatment (Extended Data Fig. 10h). Interestingly, when we evaluated the induction of RTK after DON treatment in human PDAC lines, we found that epidermal growth factor receptor (EGFR) is the predominant RTK activated in a subset of human PDAC cells (Extended Data Fig. 10i). EGFR transcript levels did not change, suggesting that regulation is post-transcriptional (Extended Data Fig. 10j). Using an inhibitor of the ErbB family resulted in a decline in basal and DON-induced levels of pERK (Extended Data Fig. 10k), implicating RTKs as major drivers of ERK induction in this context.

**Fig. 6:**
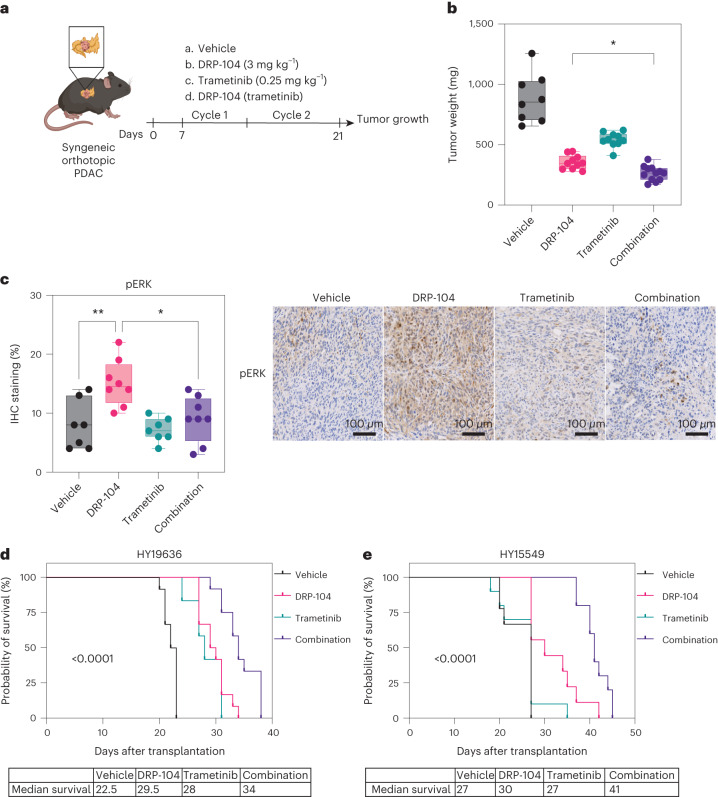
DRP-104 and MAPK inhibition increase survival in a syngeneic PDAC model. **a**, KPC-derived (HY19636) cells were injected into the pancreata of B6J mice. Mice were treated daily either with vehicle, DRP-104 (3 mg kg^−1^), trametinib (0.25 mg kg^−1^) or in combination (DRP-104 and trametinib) for two cycles (5 days on, 2 days off). Samples were collected 21 days after transplantation for downstream evaluation. **b**, Tumor weights at the end point. Vehicle, *n* = 8; DRP-104, *n* = 10; trametinib, *n* = 10; combination, *n* = 12. *P = 0.0444 (DRP-104 versus combination). Significance was determined using an ordinary one-way ANOVA with Holm-Šídák’s multiple comparisons test. **c**, Quantification of pERK levels in vehicle (*n* = 7), DRP-104 (*n* = 8), trametinib (*n* = 7) and combination (*n* = 8) treatment in B6J mice (HY19636). *P = 0.0116; **P = 0.0041. Significance was determined using an ordinary one-way ANOVA with Tukey multiple comparisons test. **d**,**e**, HY19636 (**d**) or HY15549 (**e**) cells were injected into the pancreata of B6J mice. Mice were treated daily either with vehicle (*n* = 12 biologically independent mice), DRP-104 (*n* = 12 biologically independent mice), trametinib (0.25 mg kg^−1^) (*n* = 12 biologically independent mice) or in combination (*n* = 12 biologically independent mice). Treatment continued until each mouse reached the end point criteria. A log-rank (Mantel–Cox) test indicated a *P* < 0.0001. Source data

To functionally assess whether the activation of ERK promotes resistance to DRP-104, we used the MEK inhibitor trametinib. We found that trametinib decreased ERK activation in DON-treated cells (Extended Data Fig. 10l). Moreover, inhibition of the reactive upregulation of ERK by pretreatment with trametinib further impaired the entry of glucose-derived metabolites into the TCA cycle (Extended Data Fig. 10m). To evaluate whether the combination of DRP-104 with MEK/ERK inhibitors could reduce tumor growth or increase survival, DRP-104 was tested in the presence or absence of trametinib in PDAC syngeneic models (Fig. 6a). While both DRP-104 and trametinib reduced tumor growth as single agents, we observed a more substantial decrease in tumor growth in the combination treatment (Fig. 6b). The combination treatment was effective in reducing the compensatory increase in pERK seen in the tumors treated with DRP-104 monotherapy (Fig. 6c). Consistent with these results, the combination treatment resulted in prolonged survival in the DRP-104 and trametinib combination (Fig. 6d–e).

## Discussion

Our findings suggest that broad Gln antagonism may provide additional therapeutic options for PDAC. Indeed, by using an antagonistic Gln analog, we showed that multiple Gln-dependent metabolic pathways involved in central carbon metabolism, nucleotide biosynthesis and lipid metabolism are important for the growth of PDAC cells and tumors. This is in line with other studies which demonstrated that Gln is pivotal for tumor growth. Our work further demonstrates a proof of concept that there may be an improved therapeutic index in targeting metabolism when there is preferential delivery to the tumor tissue using a pro-drug approach, such as DRP-104.

Our studies demonstrated a profound decrease in tumor growth in DRP-104-treated tumors in syngeneic PDAC models. We also observed a decrease in metastatic colonization on DRP-104 treatment, supporting the previously documented role of Gln in metastasis and EMT and therapy resistance^37,38^. Moreover, this antitumor effect was observed in both athymic and NSG mice, suggesting that in our PDAC models, the adaptive immune system is not the major driver of antitumor response. Previous work demonstrated that in vivo inhibition of Gln metabolism using DRP-104 could enhance the infiltration of CD8^+^ T cells and increase response to immunotherapy (anti-programmed cell death protein 1) in multiple other non-PDAC tumor models, probably through impairing the function of suppressive subsets of immune cells^17,26^. Interestingly, a report showed that in PDAC there is a role for T cell immunity in the response to DON^39^. However, it is important to note that this observation was made in a model in which PDAC lines were coinjected with cancer-associated fibroblasts^39^. Several reports showed that the stroma compartment of PDAC can affect the TME; therefore, the coinjection approach may alter the involvement of the adaptive immune system in this setting^40–42^. Consequently, it is possible that the involvement of the immune system in response to DON/DRP-104 in PDAC is highly context-dependent and may depend on the immunogenicity of a particular tumor and the TME.

We observed that residual tumors after DRP-104 treatment showed increased proliferation, suggesting the development of acquired resistance, and identified that DRP-104/DON treatment promotes increased activation of ERK signaling. We further demonstrated that DON-treated PDAC cells induce the activation of multiple RTKs upstream of the ERK pathway. The induction of several RTKs (for example, ErbB family, Axl) in DON-treated cells suggests that cells are coping with metabolic stress by activating multiple survival mechanisms. Interestingly, Axl was identified as an essential driver of drug resistance and nutrient scavenging through macropinocytosis in PDAC and other cancer models^43,44^. Regarding members of the ErbB family, EGFR was essential to drive macropinocytosis in a subset of PDAC under Gln deprivation^45^. Indeed, it is possible that PDAC cells functionally use Axl and ErbB family induction to drive macropinocytosis as an attempt to restore metabolic homeostasis on DON treatment. Future studies investigating the impact of broad Gln antagonism in the tumor stroma will also provide more details about potential adaptation mechanisms.

One of the limitations of this study was the lack of metabolic tools to delineate the downstream metabolic effects of DRP-104 treatment in vivo. The highly heterogenous PDAC microenvironment and the complex spatial influences in Gln use make detailed studies of metabolic changes difficult. Future studies using spatial metabolomic approaches may be informative in this regard.

As ERK signaling drives several biological processes, including survival, migration and metabolism, we explored inhibition of this pathway as a potential combination treatment. Indeed, combination treatment with DRP-104 and the MEK inhibitor trametinib reduced tumor growth and prolonged survival in syngeneic PDAC models. While survival was significantly improved in these proof-of-concept studies, we did not see evidence of complete tumor regression, suggesting that more potent combinations will be needed to ensure durable results. Whether combinations of RTK inhibitors or oncogenic KRAS inhibitors, along with DRP-104, would further improve responses is to be determined. It is also possible that DRP-104 could synergize with standard chemotherapy regimens for PDAC, such as FOLFIRINOX, as several of the components of this regimen are anti-metabolites.

In summary, our results continue to support the concept of targeting rewired metabolism in cancer and that there is a potential to achieve a favorable therapeutic index with such approaches. Because of the multiple fates of Gln in PDAC, one must broadly inhibit these pathways rather than selectively target individual pathways (such as GLS inhibition) to prevent the rapid rewiring of cellular metabolism. Future studies will continue to explore how to navigate this complex relationship between preventing metabolic rewiring and minimizing toxicity to advance critical findings into patient trials.

## Methods

The experiments in this study were performed in compliance with the Institutional Animal Care and Use Committee (IACUC) protocol no. IA16-00507; the institutional review board (IRB) of the University of New York Grossman School of Medicine (no. S17-00651) was used for the PDX-related experiments.

### Cell culture

PaTu-8988T, PANC1, MiaPaCa2 and PaTu-8902 cells were obtained from ATCC or the Deutsche Sammlung von Mikroorganismen und Zellkulturen. Primary murine PDAC cell lines (HY19636 and HY15549) were isolated from tumors from C57BL/6 (B6) genetically engineered mice (LSL-Kras^G12D^; p53^L/+^, Ptf1a^Cre+^) as described previously^46^. All cell lines were grown in 5% CO_2_ and 37 °C. All cell lines were cultured in DMEM (catalog no. 10-017-CV, Corning) with 10% FCS (catalog no. S11550H, Atlanta Biologicals) and 1% penicillin-streptomycin (catalog no. 15140122, Thermo Fisher Scientific). Cells were tested routinely for *Mycoplasma* contamination using PCR. Cell lines were authenticated by periodic fingerprinting and visual inspection; low-passage cultures were carefully maintained in a central laboratory cell bank.

### Chemicals

^13^C_5_-labeled Gln (catalog no. CLM-1822-H), alpha-^15^N Gln (catalog no. NLM-1016-PK), ^13^C_6_-labeled glucose (catalog no. CLM-1396-PK), ^15^N-labeled alanine (catalog no. NLM-454) and ^13^C_x_,^15^N_x_-labeled amino acid standard mix (catalog no. MSK-A2) were acquired from Cambridge Isotope Laboratories. *N*-acetyl-l-cysteine (catalog no. A7250) dimethylsulfoxide (DMSO), methoxyamine hydrochloride and MTBSTFA + 1% t-BDMSC and DON (catalog no. D2141) were obtained from Sigma-Aldrich. Trametinib (catalog no. 16292) and dacomitinib (pan-RTK inhibitor, catalog no.9001879) were obtained from Cayman Chemical.

### Cell proliferation and viability assays

Proliferation assays were performed at various densities depending on the growth kinetics of each line^28^. HY19636 and HY15549 were plated at 1,000 cells per well; MiaPaCa2, PANC1 and PaTu-8988T were plated at 2,000 cells per well in a 96-well plate. Cells were allowed to attach overnight. The next day, cells were treated with DON at increasing concentrations, as indicated in the figure legends. Cell growth was assessed every 12 h using Cytation C10 (Agilent Technologies) for 72 h. Confluency was derived from the object sum area divided by the total area. Every cell proliferation assay was performed with at least two technical replicates and biological replicates. For the Gln rescue assay, cells were plated at 1,000 cells per well and supplemented with the indicated Gln concentration (10 mM, 4 mM, 2 mM, 0.5 mM). DON was added the next day; cellular confluency was obtained after 7 days using Cytation C10.

### OCR

Basal OCR was measured using a Seahorse XFe96 analyzer (Agilent Technologies) as described previously^47^. PDAC lines were plated at 10,000–20,000 cells per well depending on the growth kinetics of the line in either standard DMEM or with DON for 24 h before the assay. Before the assay, the medium was removed and replaced with the XF assay medium (25 mM glucose, 4 mM Gln, no sodium bicarbonate, 5 mM HEPES); the plate was incubated for approximately 45 min at 37 °C in a non-CO_2_ incubator (Thermo Fisher Scientific). Medium was changed before the beginning of the assay; drug treatments were maintained in the XF assay medium throughout the experiment. Cell numbers were normalized to protein quantification after the assay using the DC Protein Assay Kit (Bio-Rad Laboratories). At least two independent experiments were performed for all conditions; data from 6–12 technical replicate wells for each separate experiment were averaged. Data were plotted relative to each independent group’s untreated or control condition.

### Metabolomics

For metabolite extraction, cells were washed with 0.9% NaCl prepared in high-performance liquid chromatography-grade water to remove medium and other contaminants. As described previously, cellular metabolites were collected using a methanol-water-chloroform solution^47,48^. Cellular metabolites were vortexed at 4 °C for 15 min. To separate the inorganic layers, samples were centrifuged at maximum speed for 10 min. Polar metabolites were obtained from the aqueous phase and transferred (300 µl) into polypropylene vials (catalog no. 5190-2243, Agilent Technologies) and dried down by SpeedVac (Thermo Savant, catalog no. SPD111V, Thermo Fisher Scientific). Once metabolites were dried, 20 µl methoxyamine hydrochloride (20 mg ml^−1^ in pyridine, always prepared fresh) was added and incubated for 60 min at 37 °C. Next, 20 µl MTBSTFA + 1% t-BDMSC was added and incubated for 30 min at 37 °C. Samples were analyzed using GC–MS using a gas chromatograph (7890B model, Agilent Technologies) with a DB-35ms Ultra Inert column (catalog no. 122-3832UI, Agilent Technologies) installed and associated with an Agilent 5977B mass spectrometer. The GC–MS parameters, quantification and correction for natural isotope abundances were performed as described previously^28,47,48^. For the ^13^C_6_-glucose and ^13^C_5_-Gln tracing experiments,^15^N-Gln or ^15^N-alanine cells were plated in a six-well plate at 2.0 × 10^5^ cells per well and allowed to attach overnight in DMEM. Next, cells were washed with PBS twice and cultured for 24 h in DMEM supplemented with 4 mM ^13^C_5_-Gln, 25 mM ^13^C_6_-Glucose or 1 mM ^15^N-Alanine; 10% dialyzed serum was added. For the tracing studies, cells were pretreated with DON (25 µM) overnight, medium was removed and washed with PBS. Then, cells were incubated with fresh DON and with stable isotopes for 24 h. For amino acid and metabolite profiling, metabolites were collected using an 80% methanol solution with 1 µg norvaline and labeled with amino acid standards (catalog no. MSK-A2-1.2, Cambridge Isotope Laboratories) as described elsewhere^28,47,48^. For the LC–MS analysis, cells were treated for 24 h with PBS or DON. Samples were subsequently processed by the Metabolomics Core Research Laboratory at New York University (NYU) Langone Health, as described previously^47^.

### Mice

All animal studies were approved by the NYULMC IACUC (protocol no. IA16-00507). Female B6J mice (C57BL/6J, Taconic), athymic (NCrNU, Taconic) or NSG (The Jackson Laboratory) used in this study were 8–10 weeks old and not involved in previous procedures. All mice were maintained in the animal facility of the NYU Grossman School of Medicine with access to a standard diet (PicoLab Rodent diet 20, catalog no. 5053) and water ad libitum at constant ambient temperature and a 12-h light cycle. Animal numbers were determined according to previous work^28,49^. Maximum tumor burden was limited to less than 2 cm in any direction; this metric was not exceeded in this study.

### Orthotopic xenograft experiments

Orthotopic injections of PDAC cells were conducted as described previously^28^. Briefly, mice were anesthetized with ketamine (120 mg kg^−1^) and xylazine (10 mg kg^−1^) before surgery. An incision was made in proximity to the spleen and the pancreas was carefully externalized. Cells were resuspended in 20 µl of a solution consisting of Hank’s balanced salt solution (HBSS) and Matrigel (catalog no. 356231, Corning) at a 1:1 ratio and injected into the tail of the pancreas using insulin syringes (29-gauge needle, catalog no. 324702, BD). For HY19636 and HY15549 cells, approximately 40,000 cells were injected unless indicated otherwise. For PANC1, 50,000 cells were injected into nude mice. After surgery, the incision was closed using a 3-0 coated VICRYL VIOLET suture (catalog no. J311H, Ethicon) and using the BD AutoClip Wound Closing System. Mice were treated with buprenex every 12 h after surgery for 48 h. Animals were allowed to recover from surgery for at least 7 days before starting the experimental process. Animals were randomly assigned before starting the experimental process, unless indicated otherwise. Experimental treatment started 7 days after surgery. Tumor weight was measured at the end point after mice were euthanized and tumor was collected for further analysis (for example, histology, bulk RNA-seq).

As described previously^49^, for the hemi-splenic injections, 1 × 10^5^ cells were resuspended in 100 µl HBSS and incorporated into an insulin syringe (28-gauge needle, catalog no. 329461, BD). The spleen was separated using ligating clips (catalog no. 002200, Teleflex), and cells were injected into the hemi-spleen. After injection, the splenic vein was ligated with ligating clips (catalog no. 001200, Teleflex) at the hilum of the spleen; then the hemi-spleen was removed. The peritoneum was closed with a 3-0 VICRYL VIOLET suture and the skin was closed using the BD AutoClip Wound Closing System.

For the DRP-104 studies, mice were treated daily either with vehicle (Tween-80:Ethanol:Saline 5:5:90 v/v/v) or with DRP-104 (3 mg kg^−1^) intraperitoneally for two cycles (5 days on, 2 days off) unless indicated otherwise. DRP-104 was a gift from Dracen Pharmaceuticals. DRP-104 was prepared and stored at 4 °C in the dark and used within 5 days. Trametinib was dissolved in DMSO and resuspended in a solution of 0.5% Tween-80, 0.6% 2-hydroxypropyl (catalog no. H107-5G, Sigma-Aldrich) and 0.9% saline. Animals were treated with 0.25 mg kg^−1^ daily as described previously^50^. For the hemi-splenic studies, animals were enrolled in treatment 3 days after surgery and tissue was collected 14 days after surgery. For the combination treatment (DRP-104 and trametinib), tumors were collected after two cycles (two cycles of 5 days on, 2 days off) of treatment unless indicated otherwise. For the survival studies, either treatment modality continued until the animal reached the end point criteria. The end point criteria included but were not limited to abdominal hemorrhagic ascites, severe lethargy, cachexia, weight loss, poor posture, extreme weakness or unresponsive to external stimuli according to the IACUC approved protocol (no. IA16-00507).

### In vivo metabolomics

For the in vivo metabolomics studies, HY19636 cells were orthotopically transplanted into B6J mice. Mice were treated with DRP-104 (3 mg kg^−1^) for either one or two cycles. Tumors were collected 30 min after the last DRP-104 dose; each tumor was snap-frozen in liquid nitrogen at the same time after euthanasia. Samples were subsequently processed at the Metabolomics Core Research Laboratory at NYU Langone Health. Briefly, frozen tissues were powdered with a biopulverizer (Biospec) and a fraction of the powder (approximately 10 mg) was transferred to a screw top vial for further mechanical homogenization with zircon beads using a BeadBlaster D2400 (Benchmark Scientific) at a fixed ratio of 20 mg ml^−1^ powder to extraction solvent comprising 80% LC–MS grade methanol containing isotopic internal standards (catalog no. MSK-A2-1.2, Cambridge Isotope Laboratories). Samples were homogenized for ten cycles at 30 ms and the resulting lysate was centrifuged at 21,000*g* for 3 min. Then, a fixed 450-µl volume of the metabolite supernatant was transferred to a new vial for speed vacuum concentration. The dried metabolite fraction was resolubilized in a fixed volume of 50 µl LC–MS grade water representing 1 mg of the original on-column tissue.

### In vivo PDX efficacy studies

PDX samples from individuals with PDAC were used to test the in vivo efficacy of DRP-104. Materials were obtained under the participant’s consent and approved IRB (no. S17-00651) protocol at NYU Langone Health. NSG mice (female, 8–10 weeks old) were purchased from The Jackson Laboratory. All mice were bred and maintained at the animal facility of the NYU Grossman School of Medicine. All PDX samples were developed by direct engraftment of pancreatic cancer tissue fragments from patients undergoing surgical resection or biopsy into NSG mice, expanded and viably frozen. Except for sample NYU326, all PDX samples were therapy naive. Tumor fragments were implanted subcutaneously into NSG mice and expanded by passaging in the mouse without exposing samples to in vitro conditions. PDX was passaged in NSG mice to develop treatment cohorts. Mice were randomized and enrolled into each arm (vehicle versus DRP-104) once the tumor reached 2–4 mm diameter. Mice were treated with vehicle or 3 mg kg^−1^ DRP-104. Tumor length and width were measured every 5 days using a digital caliper. Tumor volumes were calculated using the formula (L × W^2^)/2. Animals were humanely euthanized when tumors reached more than 1,000 mm^3^ or at the end of four cycles of treatment.

### PDO proliferation assays

PDO samples from individuals with PDAC were used to test the in vitro efficacy of DON. Materials were obtained under the participant’s consent and approved IRB (no. S17-00651) protocol at NYU Langone Health. All PDOs samples were developed by direct engraftment of pancreatic cancer tissue fragments from patients undergoing surgical resection or biopsy as domes in growth factor-reduced Matrigel, expanded and viably frozen at a low passage. PDOs were grown in PancreaCult Organoid Media (STEMCELL Technologies). PDOs were plated into 20 µl of growth factor-reduced Matrigel and supplemented with organoid media. PDOs were treated with DON at various doses 24 h after plating. PDO growth was assessed every 24 h using Cytation C10 for 7–10 days, depending on the growth kinetics of the PDO. Cell growth calculation was made based on the total optical density of each well.

### Histology and IHC

Tumors were fixed in 10% formalin overnight and embedded in paraffin. Paraffin sections were deparaffinized and antigens were unmasked with citrate (pH 6) and heat. Slides were incubated in 3% hydrogen peroxide and 50% methanol for 30 min and blocked in 5% goat serum and 1% BSA in Tris-buffered saline with 0.05% Tween 20 (TBST) for 30 min at room temperature. Primary antibodies were diluted in blocking buffer and applied overnight at 4 °C, then developed using the VECTASTAIN Elite ABC-HRP kit (catalog no. PK-6100, Vector Laboratories) and DAB Substrate Kit (catalog no. SK-4100, Vector Laboratories). Slides were counterstained with hematoxylin (catalog no. H-3401, Vector Laboratories). Sections were dehydrated and mounted in Fisher Chemical Permount Mounting Medium (catalog no. SP15-100, Thermo Fisher Scientific) and allowed to dry overnight before slide imagining. The primary antibody was diluted in blocking buffer and added to sections at 4 °C overnight. Quantification of IHC was performed using the Aperio ImageScope software (Leica Biosystems); non-tumor and necrotic areas were not included in the analysis as described previously^51^. Briefly, we measured the percentage (%) of positivity resulting from the positive signal divided by the total area analyzed (Positive/AreaTotal).

To evaluate the role of CAF, 5-μm sections of paraffin-embedded tissue were stained with Akoya Biosciences Opal multiplex automation kit reagents unless stated otherwise. Automated staining was performed on a Leica Bond RX autostainer. The protocol was performed according to the manufacturer’s instructions with the following antibodies: CK19 (1:500 dilution, catalog no. MABT913, Merck Millipore), aSMA (1:4,000 dilution, catalog no. ab5694, Abcam), podoplanin monoclonal antibody (1:4,000 dilution, catalog no. 145381-82, eBioscience), PDGF R alpha (1:100 dilution, catalog no. AF1062, R&D Systems). Briefly, all slides underwent sequential epitope retrieval with the Leica Biosystems epitope retrieval 1 or 2 solution (citrate-based, pH 6.0, catalog no. AR9961; EDTA-based, pH9, catalog no. AR9640), primary and secondary antibody incubation and tyramide signal amplification with Opal fluorophores (catalog no. FP1487001KT, Akoya Biosciences). Primary and secondary antibodies were removed during the epitope retrieval steps while fluorophores were covalently attached to the epitope. Slides were mounted with ProLong Gold Antifade Mountant (catalog no. P36935, Thermo Fisher Scientific). Semiautomated image acquisition was performed on a Vectra Polaris multispectral imaging system. The inForm v.2.6 software from Akoya Biosciences was used for spectral unmixing and image analysis. To quantify the CAF subtypes, HALO (v.3.6, Indica Labs) was used. Briefly, a training algorithm was first used to identify nuclei (4,6-diamidino-2-phenylindole, nuclei segmentation AI module) on different samples during the training process. Then, the Highflex FL module algorithm (v.4.2.5, Indica Labs) was used to classify cells as CK19^+^, podoplanin^+^, aSMA^+^ and PDGFR^+^ using the phenotyper segmentation module. Each classification was reviewed for different samples during the training iterations. CAF subtypes were identified as pan-CAF (CK19^−^, podoplanin^+^); CK19^−^, podoplanin^+^, aSMA^+^, PDGFR^−^ and CK19^−^, podoplanin^−^, aSMA^+^, PDGFR^+^. The thresholds for the classification was based only on high-expressing cells. Hematoxylin and eosin, Masson trichrome staining, slide scanning and Opal multiplexing were performed by the Experimental Pathology Research Laboratory at the NYU Grossman School of Medicine.

### Western blot and antibodies

Whole-cell protein lysates were collected using radioimmunoprecipitation assay buffer (catalog no. 20-188, Sigma-Aldrich) supplemented with protease (Roche) and phosphatase (Roche) inhibitor cocktails. Protein lysates were separated on 4–20% gradient gels (catalog no. 4561096, Bio-Rad Laboratories) and transferred to polyvinylidene fluoride membranes (catalog no. IPVH00010, Merck Millipore) using transfer buffer (Tris-glycine) and 10% methanol. Membranes were blocked with 3% BSA (catalog no. A2058, Sigma-Aldrich) for at least 1 h at room temperature. Primary antibodies were incubated overnight at 4 °C at the following dilutions: ERK2 (1:1,000, catalog no. sc-1647, Santa Cruz Biotechnology); phospho-p44/42 MAPK (ERK1/2) (1:1,000, catalog no. 4376, Cell Signaling Technology); DUSP6 (1:1,000, catalog no. A2D4, Abcam); anti-*O*-linked *N*-acetylglucosamine (1:1,000, catalog no. ab2739, Abcam); cleaved Caspase-3 (1:1,000, catalog no. 9664, Cell Signaling Technology); ATF4 (1:1,000, catalog no. 11815, Cell Signaling Technology); phospho-eIF2α (Ser51) (1:1,000, catalog no. 3398, Cell Signaling Technology); Ki67 (1:500, catalog no. 16667, Abcam); GLUL (1:1,000, catalog no. 228590, Abcam); CK19 (1:100, catalog no. MABT913, Merck Millipore); anti-mouse IgG, horseradish peroxidase (HRP)-linked (1:5,000, catalog no. 7076 S, Cell Signaling Technology); anti-rabbit IgG, HRP-linked (1:5,000, catalog no. 7074 S, Cell Signaling Technology). For LI-COR imaging, IRDye 800CW goat anti-rabbit IgG (1:10,000, catalog no. 926-32211, LI-COR Biosciences) and IRDye 680RD goat anti-mouse IgG (1:10,000, catalog no. 926-68070, LI-COR Biosciences) secondary antibodies were used. Membranes were washed at least three times (15 min each) with TBST and incubated with the appropriate HRP-conjugated secondary antibody (1:5,000, catalog no. 7045S or 7076S, Cell Signaling Technology) for 1 h at room temperature; the enhanced chemiluminescence detection system was added (catalog no. 1705061, Bio-Rad Laboratories). Images were obtained with the ChemiDoc (Bio-Rad Laboratories) or Odyssey CLx (LI-COR Biosciences) imager. Each immunoblot was repeated at least twice.

### Phospho-RTK array kit

The phosphorylation status of RTK was obtained using the Profiler Mouse Phospho-RTK Array Kit (catalog no. ARY014, R&D Systems) and Proteome Profiler Human Phospho-RTK Array Kit (catalog no. ARY001B, R&D Systems). Assays were performed according to the manufacturer’s instructions for each individual batch.

### Bulk RNA-seq

Total RNA from cell cultures and tumors was obtained using TRIzol (catalog no. 15596026, Thermo Fisher Scientific) and PureLink RNA Mini Kit (catalog no. 12183025) according to the manufacturer’s instructions. Bulk RNA-seq libraries, raw data collection (FASTQ), alignment (HISAT2), mapping and differential expression analysis (DESeq2) were performed according to the manufacturer’s instructions by Novogene^52^. Logarithmic transformation (log_2_) of fragments per kilobase of transcript per million fragments mapped was used as input for GSEA^53^.

### Statistics and reproducibility

All data were analyzed using Prism 9.0 (GraphPad Software). Results are expressed as the mean and s.e.m. unless otherwise indicated in the figure legend. The experiments were not randomized, and the investigators were not blinded to allocation during the experiments and outcome assessment. No statistical method was used to predetermine sample size. No data were excluded from the analyses. For the in vivo studies, before treatment initiation, mice bearing tumors were randomized. Tumor size was not measured at the time of randomization. For the tumor growth studies, the data shown represent independent experiments with biological replicates. For the in vitro studies, including the growth curves, Seahorse analysis and western blots were performed at least twice; the representative data of one experiment was presented. For the LC–MS and GC–MS, experiments were repeated twice with at least three biological replicates. IHC images represent a randomly selected image of a single biological sample. For all experiments, data distribution was assumed to be normal, but this was not formally tested. A Student’s two-tailed unpaired *t*-test was used for the experiments with two groups unless otherwise indicated. For multiple group comparisons, a one-way or two-way ANOVA was performed, followed by a Tukey or Holm-Šídák test unless otherwise indicated in the figure legend. The box plots extend from the 25th to 75th percentile. The whiskers represent the smallest to largest values. The line in the middle of the box represents the median. Data were considered significant if *P* < 0.05; exact values are shown in each figure legend or in the source data file.

### Reporting summary

Further information on research design is available in the Nature Portfolio Reporting Summary linked to this article.

### Supplementary information

Reporting Summary

### Source data

Source Data Fig. 1 Data source and statistics.

Source Data Fig. 2 Data source and statistics.

Source Data Fig. 3 Data source and statistics.

Source Data Fig. 4 Data source and statistics.

Source Data Fig. 5 Data source and statistics.

Source Data Fig. 6 Data source and statistics.

Source Data Extended Uncropped gels.

Source Data Extended Data Fig. 1 Data source and statistics.

Source Data Extended Data Fig. 2 Data source and statistics.

Source Data Extended Data Fig. 3 Data source and statistics.

**Extended Data Fig. 4:**
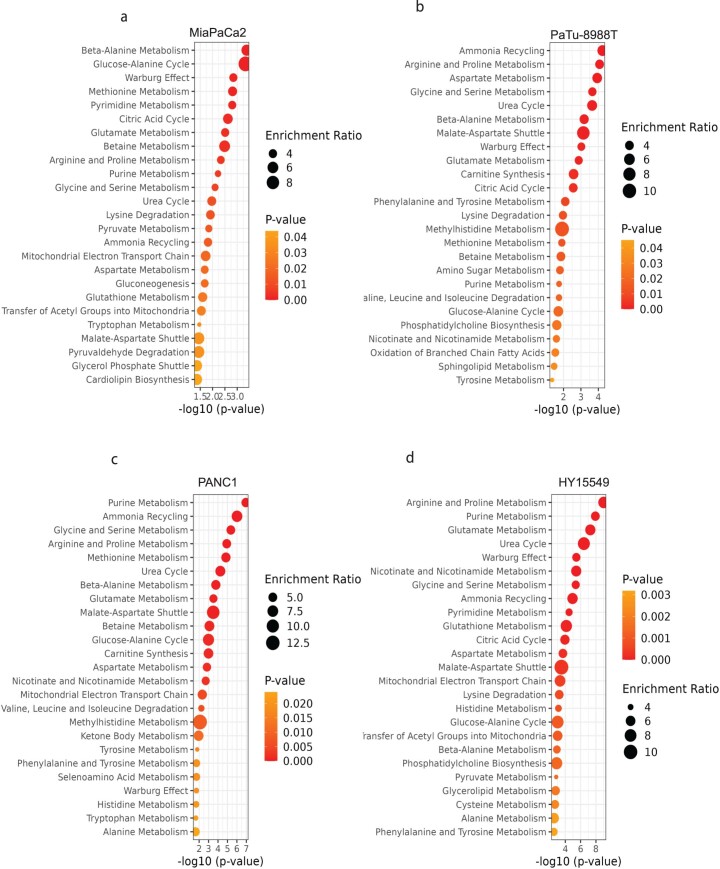
DON treatment globally disrupted central carbon and nucleotide metabolism. Metabolite Set Enrichment Analysis of various PDAC lines (**a**) MiaPaCa2, (**b**)PaTu-8988T, (**c**)PANC1, and (d)HY15549. Values were obtained from LCMS analysis, treated with DON (25 µM, 24 hours). The input for Metabolite Set Enrichment Analysis were based on P Value < 0.05. Significance was determined using the Wald test (DESeq2) corrected for multiple comparisons. All values are available in the source data. Source data

Source Data Extended Data Fig. 4 Data source and statistics.

**Extended Data Fig. 5:**
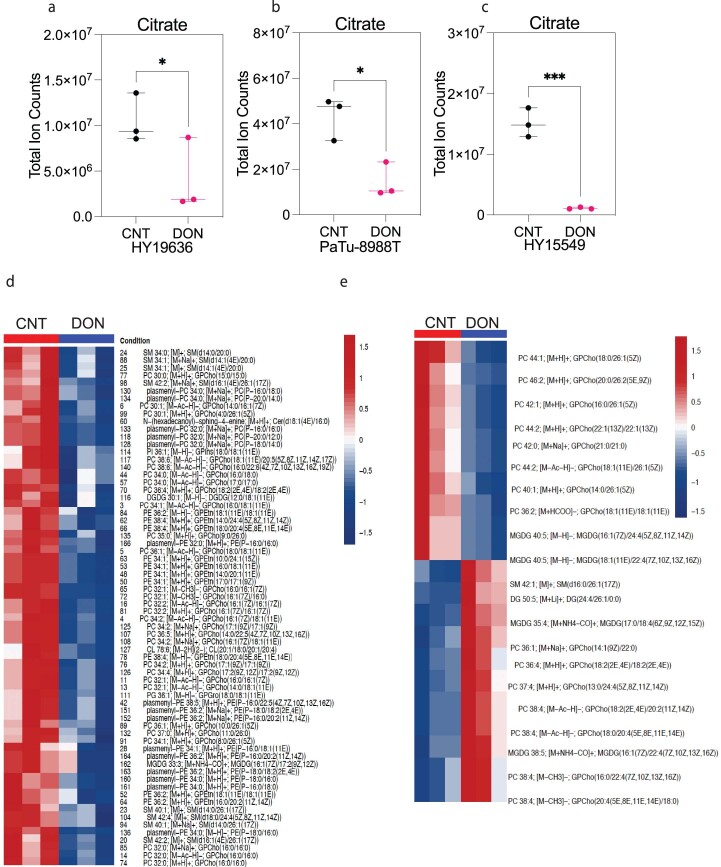
DON treatment globally disrupts lipid metabolism. (**a**)Total ion counts of citrate in HY19636 cells treated with DON (25 µM) for 24 hours. VEH, n = 3; DRP-104, n = 3; P value: 0.0413 (*). Data obtained through GCMS normalized to internal standard (norvaline) and cell counts. Data displayed as mean ± s.e.m. and significance were determined using unpaired t-test (two-tailed). (**b**)Total ion counts of citrate in PaTu-8988T cells treated with DON (25 µM) for 24 hours. VEH, n = 3; DRP-104, n = 3; P value: 0.0143 (*). Data obtained through GCMS normalized to internal standard (norvaline) and cell counts. Data displayed as mean ± s.e.m and significance were determined using unpaired t-test two-tailed. (**c**)Total ion counts of citrate in HY15549 cells treated with DON (25 µM) for 24 hours. VEH, n = 3; DRP-104, n = 3; P value: 0.0005 (***) Data obtained through GCMS normalized to internal standards and cell counts. Data displayed as mean ± s.e.m. and significance were determined using unpaired t-test two-tailed. (**d**) HY15549 and (**e**)PaTu-8988T cells were treated with DON for 24 hours. For both cell lines, VEH, n = 3; DRP-104, n = 3. LCMS was performed after scaling the lipid extraction to a measured aliquot ( ~ 5e6/mL) for each of the samples. Lipid peak intensities were extracted according to a library of m/z values and retention times developed with standards. LipidBLAST was used to identify lipid species and features. Heatmap was generated by using the significant metabolites (p < 0.05). Significance was determined using the Wald test (DESeq2) corrected for multiple comparisons. Complete dataset and statistics are available in the source data. Source data

Source Data Extended Data Fig. 5 Data source and statistics.

**Extended Data Fig. 6:**
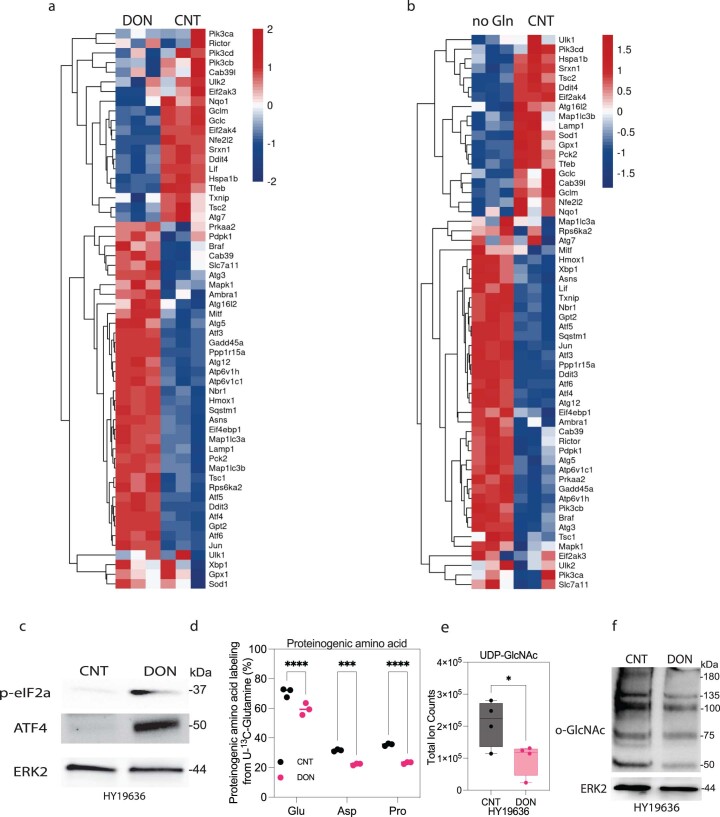
Stress-related pathways are activated upon DON treatment. (**a**) Bulk RNAseq demonstrated increased expression of genes in various stress-related pathways after 24 hours of DON treatment (25 µM) in HY19636 cells. (**b**) Bulk RNAseq demonstrated increased expression of genes in various stress-related pathways after 24 hours of glutamine deprivation in HY19636 cells. (**c**) Immunoblot of p-eIF2α, ATF4 and ERK2 in DON-treated cells (25 µM) (HY19636). The image is representative of two independent experiments. (**d**) Percentage of contribution of [U-^13^C_5_]-Glutamine to proteinogenic amino acids (for example, glutamate, aspartate, and proline) in DON-treated cells (HY19636) for 24 hours. VEH, n = 3; DRP-104, n = 3. P values: <0.0001(****); 0.0004 (***); < 0.0001(****). Data was obtained through GCMS and represented as percentages. Data displayed as mean ± s.e.m. and significance were determined using ordinary one-way ANOVA using Šídák’s multiple comparisons test. (**e**) Total levels of UDP-GlcNAc detected via LC-MS in DON-treated cells (25 µM). VEH, N = 4; DRP-104, n = 4. P value: 0.0434 (*).Total ion counts were normalized to cell number (HY19636). Data displayed as mean ± s.e.m. and significance were determined was unpaired t-test (two-tailed). The box plots extend from the 25th to 75th percentiles. The whiskers represent the smallest to largest values. The line in the middle of the box is plotted at the median. (**f**) Immunoblot of O-GlcNAc and ERK2 (loading control) in DON-treated (25 µM) cells (HY19636). The image is representative of two independent experiments. Source data

Source Data Extended Data Fig. 6 Data source and statistics.

**Extended Data Fig. 7:**
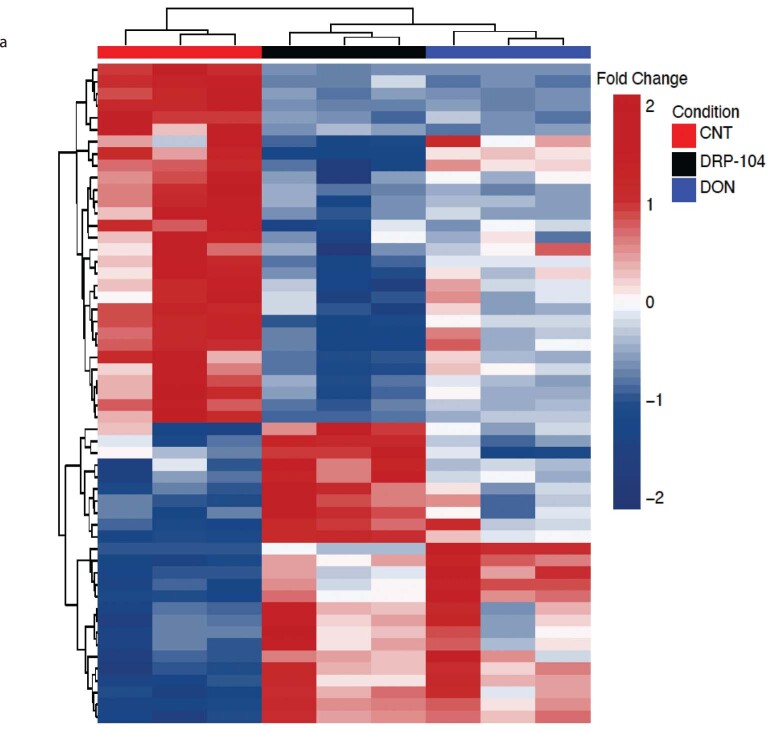
DRP-104 creates a metabolic crisis in PDAC similar to DON. HY19636 were treated with DRP-104 (25 µM) and DON (25 µM) for 24 hours. (**a**)Heatmap was generated by unsupervised clustering using the significant metabolites (p-value < 0.05) from the comparison of CNT vs DON and CNT vs DRP-104. Significance was determined using the Wald test (DESeq2) corrected for multiple comparisons. Source data

Source Data Extended Data Fig. 7 Data source and statistics.

**Extended Data Fig. 8:**
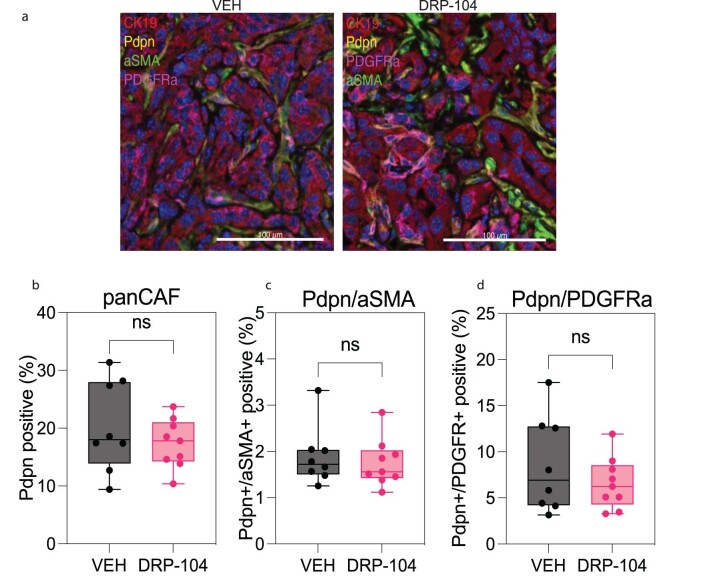
Assessment of cancer-associated fibroblasts upon DRP-104. **a**) Representative multispectral imaging of PDAC cell lines (CK19-, red), panCAFs (Pdpn, yellow), aSMA (green), PDGFR (magenta) in DRP-104 treated (n = 9) and VEH (n = 8) tumors. (**b**) Quantification of pan-CAF (Pdpn-positive) abundance in DRP-104 treated and control tumors. VEH, n = 8; DRP-104, n = 9. Unpaired t-test (two-tailed) showed no significance P value: 0.3411. The box plots extend from the 25th to 75th percentiles. The whiskers represent the smallest to largest values. The line in the middle of the box is plotted at the median. (**c**) Quantification of Pdpn-positive, aSMA-positive populations in DRP-104 treated and VEH tumors. VEH, n = 8; DRP-104, n = 9. Unpaired t-test (two-tailed) showed no significance P value: 0.6294. The box plots extend from the 25th to 75th percentiles. The whiskers represent the smallest to largest values. The line in the middle of the box is plotted at the median. (**d**) Quantification of Pdpn-positive, PDGFR-positive populations in DRP-104 treated and VEH tumors. VEH, n = 8; DRP-104, n = 9. Unpaired t-test (two-tailed) showed no significance P value: 0.3358. The box plots extend from the 25th to 75th percentiles. The whiskers represent the smallest to largest values. The line in the middle of the box is plotted at the median. Source data

Source Data Extended Data Fig. 8 Data source and statistics.

**Extended Data Fig. 9:**
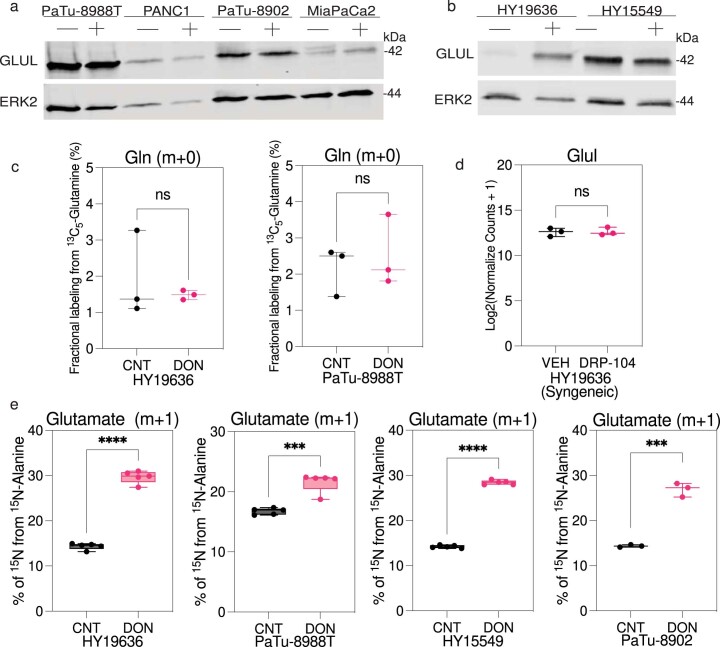
Alanine supports metabolism in DON-treated cells. (**a**) Immunoblot analysis for GLUL and ERK2 (loading control) in PaTu-8988T, PANC1, PaTu-8902 and MiaPaCa2 cells treated with DON (25 µM) for 72 hours. The image is representative of two independent experiments. (**b**)Immunoblot analysis for GLUL and ERK2 (loading control) in HY19636 and HY15549 cells treated with DON (25 µM) for 72 hours. The image is representative of two independent experiments. (**c**) HY19636 and PaTu-8988T cells were treated with DON (25 µM) for 72 hours. Then, cells were exposed to U-^13^C_5_-Glutamine for 24 hours. Data plotted as percentage of m + 0 (unlabeled) fraction. VEH, n = 3; DON, n = 3. Data displayed as mean ± s.e.m. Unpaired t-test (two-tailed) showed no significance P value: 0.5613 (HY19636) and 0.6234 (PaTu-8988T). (**d**) GLUL expression upon DRP-104 treatment in syngeneic tumors (HY19636) at endpoint. Data obtained from bulkRNA-seq studies. Unpaired t-test (two-tailed) showed no significance P value: 0.9019. (**e**) Percentage of incorporation of ^15^N-Alanine into glutamate after DON treatment in HY19636, PaTu-8988T, HY15549 and PaTu-8902 cells (25 µM, 24 hours). For HY1936, VEH, n = 5; DON, n = 5. P value: <0.0001 (****). For PaTu-8988T, VEH, n = 5; DON, n = 5. P value: 0.0002 (***); For HY15549, VEH, n = 5; DON, n = 5. P value: <0.0001 (****). For PaTu-8902, VEH, n = 3; DON, n = 3. P value: 0.0001 (***). Data displayed as mean ± s.e.m. and significance were determined was unpaired t-test (two-tailed). Source data

Source Data Extended Data Fig. 9 Data source and statistics.

**Extended Data Fig. 10:**
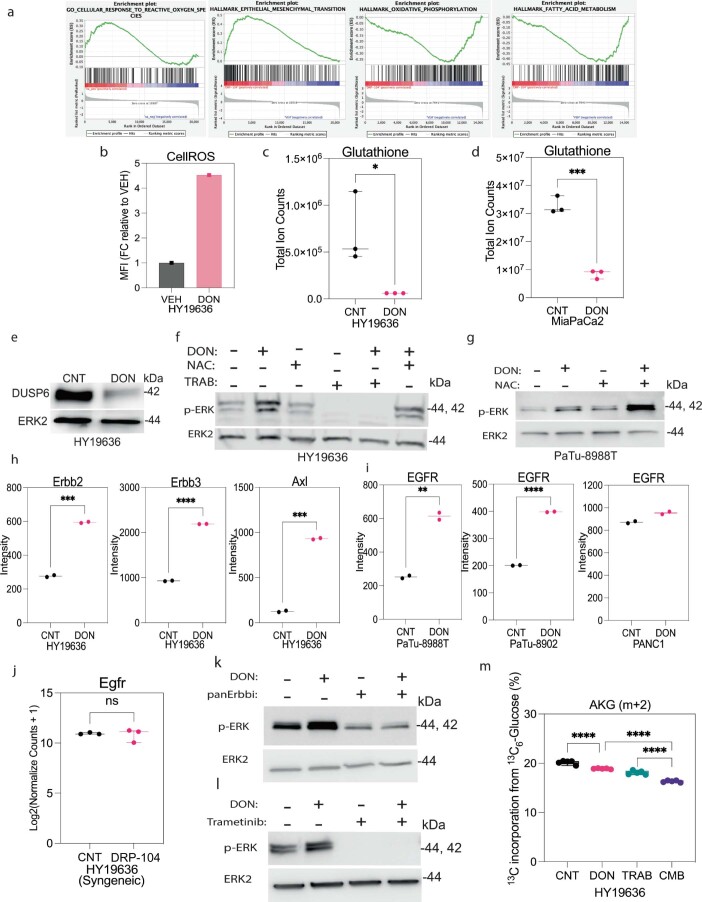
Multiple inputs can lead to MAPK activation in response to DON treatment. (**a**) Gene set enrichment analysis (GSEA) of transcripts between DRP-104 treated and control tumors demonstrated enrichment in ROS, EMT, OXPHOS, fatty acid Metabolism. (**b**) Mean Fluorescent Intensity (MFI) of CellROS in DON versus control cells measured by FACS in HY19636 cells. (**c**) Total levels of glutathione detected via LC-MS in DON-treated cells in HY19636. Total abundance was represented as fold change relative to control. VEH, n = 3; DRP-104, n = 3. P value: 0.0413 (*).Data displayed as mean ± s.e.m. and significance were determined was unpaired t-test two-tailed. (**d**) Total levels of glutathione detected via LC-MS in DON-treated cells in MiaPaCa2. Total abundance was represented as fold change relative to control. VEH, n = 3; DRP-104, n = 3. P value: 0.0002 (***). Data displayed as mean ± s.e.m. and significance were determined was unpaired t-test two-tailed. (**e**) Immunoblot of DUSP6 and ERK2 (loading control) in DON-treated cells in HY19636 cells. The image is representative of three independent experiments. (**f**) Immunoblot of p-ERK and ERK2 (loading control) in cells treated with DON, NAC (10 mM), trametinib or the combination for 24 hours in HY19636 cells. The image is representative of three independent experiments. (**g**) Immunoblot of p-ERK and ERK2 (loading control) in cells treated with DON, NAC (10 mM), or the combination for 24 hours in PaTu-8988T cells. The image is representative of three independent experiments. (**h**) Quantification of phospho-RTK array in HY19636 cells upon DON treatment (25 µM, 72 hours). Data were quantified by ImageJ. VEH, n = 2; DON, n = 2. P value for Erbb2 0.0004 (***); P value for Erbb3 < 0.0001 (****); P value for Axl 0.0002 (***). Representative experiment. Experiment was repeated two times with similar results. Data displayed as mean ± s.e.m. and significance were determined was unpaired t-test two-tailed. (**i**) Quantification of phospho-RTK array (EGFR) in PaTu-8988T, PATu-8902 and PANC1 cells upon DON treatment (25 µM, 72 hours). Data were quantified by ImageJ. VEH, n = 2; DON, n = 2. P value for PaTu-8988T: 0.0038 (**). P value for PaTu-8902: <0.0001 ****. P value for PANC1: 0.002. Representative experiment. Experiment was repeated two times with similar results. Data displayed as mean ± s.e.m. and significance were determined was unpaired t-test two-tailed. (**j**) Relative mRNA levels of Egfr in DRP-104 treated tumors. VEH, n = 3; DRP-104, n = 3. P value: 0.7406 (ns). Data displayed as mean ± s.e.m. and significance were determined was unpaired t-test two-tailed. (**k**) Immunoblot of p-ERK and ERK2 (loading control) in cells treated with DON, pan-RTK inhibitor or the combination for 24 hours. The image is representative of two independent experiments. (**l**) Immunoblot of p-ERK and ERK2 (loading control) in cells treated with DON, trametinib or the combination for 24 hours. The image is representative of three independent experiments. (**m**) Percentage of incorporation of ^13^C_6_-Glucose to alpha-ketoglutarate (AKG) (m + 2) in cells pre-treated with trametinib with or without DON for 24 hours in HY19636.VEH, n = 6; DON, n = 6. P values: <0.0001 (****); 0.0031(**). Data displayed as mean ± s.e.m. and significance determined by ordinary one-way ANOVA using Tukey’s multiple comparisons test. The box plots extend from the 25th to 75th percentiles. The whiskers represent the smallest to largest values. The line in the middle of the box is plotted at the median. Source data

Source Data Extended Data Fig. 10 Data source and statistics.

## Data Availability

The RNA-seq data that support the findings of this study have been deposited in the Gene Expression Omnibus (GEO) under accession no. GSE236225. The processed metabolomic data (GC–MS and LC–MS) generated in this study are provided in the source data. The LC–MS data have been deposited in the National Metabolomics Data Repository (https://www.metabolomicsworkbench.org/) under studies ID: ST002847, ST002853 and ST002855. All other data supporting the findings of this study are available from the corresponding author upon reasonable request. Source data are provided with this paper.

## Code availability

Metabolyze is available at https://github.com/DrewRJones/Metabolyze_Public.git under a Massachusetts Institute of Technology (MIT) license and requires Python v.3.6.5 and R v.3.5.1. The GC–MS analysis script is available at https://github.com/Sethjparker/IntegrateNetCDF_WithCorrect under an MIT license and requires MATLAB R2016 or higher.
