# Supplementary material for: Targeting pancreatic cancer metabolic dependencies through glutamine antagonism
Source: Nat Cancer. 2023 Oct 9;5(1):85–99. doi: 10.1038/s43018-023-00647-3 (PMC10824664; doi:10.1038/s43018-023-00647-3)
Supplement: Supplementary file 1 — Reporting Summary [file 43018_2023_647_MOESM1_ESM.pdf]

nature portfolio

Corresponding author(s): Alec C. Kimmelman

Last updated by author(s): 07262023

Reporting Summary

Nature Portfolio wishes to improve the reproducibility of the work that we publish. This form provides structure for consistency and transparency in reporting. For further information on Nature Portfolio policies, see our Editorial Policies and the Editorial Policy Checklist.

Statistics

For all statistical analyses, confirm that the following items are present in the figure legend, table legend, main text, or Methods section.

n/a	Confirmed	
☐	☒	The exact sample size (*n*) for each experimental group/condition, given as a discrete number and unit of measurement
☐	☒	A statement on whether measurements were taken from distinct samples or whether the same sample was measured repeatedly
☐	☒	The statistical test(s) used AND whether they are one- or two-sided<br>*Only common tests should be described solely by name; describe more complex techniques in the Methods section.*
☒	☐	A description of all covariates tested
☒	☐	A description of any assumptions or corrections, such as tests of normality and adjustment for multiple comparisons
☐	☒	A full description of the statistical parameters including central tendency (e.g. means) or other basic estimates (e.g. regression coefficient) AND variation (e.g. standard deviation) or associated estimates of uncertainty (e.g. confidence intervals)
☐	☒	For null hypothesis testing, the test statistic (e.g. *F*, *t*, *r*) with confidence intervals, effect sizes, degrees of freedom and *P* value noted<br>*Give P values as exact values whenever suitable.*
☒	☐	For Bayesian analysis, information on the choice of priors and Markov chain Monte Carlo settings
☒	☐	For hierarchical and complex designs, identification of the appropriate level for tests and full reporting of outcomes
☒	☐	Estimates of effect sizes (e.g. Cohen's *d*, Pearson's *r*), indicating how they were calculated

Our web collection on statistics for biologists contains articles on many of the points above.

Software and code

Policy information about availability of computer code

Data collection	ChemiDoc (Biorad); Odyssey CLx (Licor Bioscience); Vectra® Polaris multispectral imaging system; CytationC10 (Agilent), Agilent 5977B mass spectrometer.
Data analysis	InForm® (Akoya Biosciences, version 2.6); HALO (Indica Labs, v.3.6) ; Graphpad Prism 9.0 (Graph Pad); Python 3.6.5; Matlab R2016 and R 3.5.1. For Gene Set Enrichment Analysis was conducted using GSEA software (version 3.0) with gene sets derived from the KEGG pathway database in the MSigDB collections. Statistical analysis was performed using Graphpad Prism (GraphPad Software, version 9). Gen5 Cytation Software (v3.11).<br><br>Metabolyze is available at https://github.com/DrewRJones/Metabolyze_Public.git (Accessed on July 10, 2021) under MIT license.<br>GC-MS analysis script is available at https://github.com/Sethjparker/IntegrateNetCDF_WithCorrect under MIT license.

For manuscripts utilizing custom algorithms or software that are central to the research but not yet described in published literature, software must be made available to editors and reviewers. We strongly encourage code deposition in a community repository (e.g. GitHub). See the Nature Portfolio guidelines for submitting code & software for further information.

Data

Policy information about availability of data

All manuscripts must include a data availability statement. This statement should provide the following information, where applicable:

- Accession codes, unique identifiers, or web links for publicly available datasets
- A description of any restrictions on data availability
- For clinical datasets or third party data, please ensure that the statement adheres to our policy

RNA-Seq data that support the findings of this study have been deposited in the Gene Expression Omnibus (GEO) under accession codes GSE236225.The processed metabolomic data (GC-MS and LC-MS) generated in this study are provided in the Source Data file. Source data for Fig. 1-5 and Extended Data Fig. 1-10 have been provided as Source Data files. Further information and requests for reagents may be directed to and will be fulfilled by the corresponding author Alec C. Kimmelman (Alec.Kimmelman@nyulangone.org) on reasonable request.

Research involving human participants, their data, or biological material

Policy information about studies with human participants or human data. See also policy information about sex, gender (identity/presentation), and sexual orientation and race, ethnicity and racism.

Reporting on sex and gender	Sex of patient-derived xenografts<br>NYU341: Female<br>NYU559:Male<br>NYU280: Male<br>NYU338: Male<br>NYU345: Male
Reporting on race, ethnicity, or other socially relevant groupings	No report on race, ethnicity, or other socially relevant groupings was used in this study
Population characteristics	No population characteristics were used in this study.
Recruitment	No population recruitment was used in this study.
Ethics oversight	For this study All the work for this study was approved by the Institutional Animal Care and Use Committee (NYULMC-IACUC) protocol #IA16-00507. For PDX-related experiments an Institutional Review Board (S17-00651) was used. For PDX-related experiments, the study was approved by the NYU School of Medicine Institutional Review Board and the Ethics Committee. The pancreas samples were obtained from patients who signed the universal consent form for tissue collection for research studies.

Note that full information on the approval of the study protocol must also be provided in the manuscript.

Field-specific reporting

Please select the one below that is the best fit for your research. If you are not sure, read the appropriate sections before making your selection.

☒ Life sciences ☐ Behavioural & social sciences ☐ Ecological, evolutionary & environmental sciences

For a reference copy of the document with all sections, see nature.com/documents/nr-reporting-summary-flat.pdf

Life sciences study design

All studies must disclose on these points even when the disclosure is negative.

Sample size	As described in Yamamoto, et al. Nature, 2020. No sample size calculation was done either for in vitro or in vivo studies. For in vivo studies, sample sizes were determined based on our preliminary experiments. In our experience, n = 5-10 mice per group is sufficient to detect meaningful biological differences with good reproducibility.
Data exclusions	No data exclusion
Replication	To replicate our biologically meaningful findings, experiments were repeated independently. This is indicated in the associated figure legend for each result. We also utilized orthogonal approaches to validate our findings (e.g., different cell lines, orthotpic versus metastasis model; idifferent mouse backgrounds).
Randomization	Prior to treatment initiation, mice bearing tumours were randomized. Tumour sizes were not measured at the time of randomization.<br><br>Treatment for Seahorse OCR/ECAR, metabolomics, growth curves were not randomized to maintain consistent well assignments between independent experiments; however, cell plating for these assays was randomized. To control for unexpected covariates for Seahorse OCR/

ECAR experiments, multiple independent experiments were conducted. Sample acquisition by GC-MS or LC-MS was randomized to control for instrument bias and unexpected covariates.

Blinding

Blinding was not performed in mouse experiments. The investigator needed to know the treatment groups in order to perform the study. Tumour weights (an objective measurement) were carried out only at the study endpoints after mice were euthanized and tumours were harvested.

In vitro studies were not blinded because experimental and surrogate counting wells needed to be assigned and treated similarly during treatment.

Reporting for specific materials, systems and methods

We require information from authors about some types of materials, experimental systems and methods used in many studies. Here, indicate whether each material, system or method listed is relevant to your study. If you are not sure if a list item applies to your research, read the appropriate section before selecting a response.

Materials & experimental systems

n/a	Involved in the study	
☐	☒	Antibodies
☐	☒	Eukaryotic cell lines
☒	☐	Palaeontology and archaeology
☐	☒	Animals and other organisms
☒	☐	Clinical data
☒	☐	Dual use research of concern
☒	☐	Plants

Methods

n/a	Involved in the study	
☒	☐	ChIP-seq
☐	☒	Flow cytometry
☒	☐	MRI-based neuroimaging

Antibodies

Antibodies used

ERK2 (Santa Cruz, sc-1647, 1:1,000); Phospho-p44/42 MAPK (ERK1/2) (CST, 4376, 1:1,000); DUSP6 (Abcam, A2D4, 1:1,000); Anti-O-Linked N-Acetylglucosamine antibody [RL2] (Abcam, ab2739, 1:1000); Cleaved Caspase-3 (CST, 9664, 1:1,000); ATF4 (CST, 11815, 1:1,000); Phospho-eIF2α (Ser51) (CST, 3398, 1:1,000); Ki67 (Abcam,16667, 1:500); GLUL (Abcam, 228590, 1:1,000); , Anti-mouse IgG, HRP-linked Antibody (CST, 7076S, 1:5,000), Anti-rabbit IgG, HRP-linked Antibody (CST, 7074S, 1:5,000). For LICOR imaging, IRDye® 800CW Goat anti-Rabbit IgG Secondary Antibody (Licor, 926-32211, 1:10,000); IRDye® 680RD Goat anti-Mouse IgG Secondary Antibody (Licor, 926-68070, 1:10,000).

For IHC or multiplexing:
CK19 (Millipore, MABT913, 1:100)
Mouse PDGF R alpha Antibody (R&D, AF1062, 1:100)
Podoplanin Monoclonal Antibody (NZ-1.3), eBioscience™ (1:4,000)
Anti-alpha smooth muscle Actin antibody (ab5694): (1:4,000)

Secondary HRP Polymer; Vendor/Cat#:
Goat-on-Rodent HRP Polymer, Biocare GHP516H
Rat-HRP-Polymer 1-Step, Biocare BRR4016H
Peroxidase-conjugated Anti-Syrian Hamster IgG, Jackson Labs, 307-036-003
Rabbit-on-Rodent HRP Polymer, Biocare RMR622L

OPAL Fluor:
520, Akoya FP1487001KT
690, Akoya FP1497001KT
570, Akoya FP1488001KT
620, Akoya FP1495001KT

Validation

All antibodies used are commercially available and validated by the manufacturers.

Phospho-p44/42 MAPK (ERK1/2): https://www.cellsignal.com/products/primary-antibodies/phospho-p44-42-mapk-erk1-2-thr202-tyr204-20g11-rabbit-mab/4376

C-Cleaved Caspase-3 (Asp175) (5A1E) Rabbit mAb #9664: https://www.cellsignal.com/products/primary-antibodies/cleaved-caspase-3-asp175-5a1e-rabbit-mab/9664

ATF-4 (D4B8) Rabbit mAb #11815: https://www.cellsignal.com/products/primary-antibodies/atf-4-d4b8-rabbit-mab/11815

Phospho-eIF2α (Ser51) (D9G8) XP® Rabbit mAb #3398: https://www.cellsignal.com/products/primary-antibodies/phospho-eif2a-ser51-d9g8-xp-rabbit-mab/3398

Anti-mouse IgG, HRP-linked Antibody #7076: https://www.cellsignal.com/products/secondary-antibodies/anti-mouse-igg-hrp-linked-antibody/7076?site-search-type=Products&N=4294956287&Ntt=7076s&fromPage=plp&_requestid=452578

Anti-rabbit IgG, HRP-linked Antibody #7074: https://www.cellsignal.com/products/secondary-antibodies/anti-rabbit-igg-hrp-linked-antibody/7074?site-search-type=Products&N=4294956287&Ntt=7074s&fromPage=plp&_requestid=452610

Anti-DUSP6 antibody [A2D4] (ab238512): https://www.abcam.com/products/primary-antibodies/dusp6-antibody-a2d4-ab238512.html

Anti-O-Linked N-Acetylglucosamine antibody [RL2] (ab2739): https://www.abcam.com/products/primary-antibodies/o-linked-n-acetylglucosamine-antibody-rl2-ab2739.html

Recombinant Anti-Ki67 antibody [SP6] (ab16667): https://www.abcam.com/products/primary-antibodies/ki67-antibody-sp6-ab16667.html

Anti-Glutamine Synthetase antibody (ab228590): https://www.abcam.com/products/primary-antibodies/glutamine-synthetase-antibody-ab228590.html

IRDye® 800CW Goat anti-Rabbit IgG Secondary Antibody: https://www.licor.com/bio/reagents/irdye-800cw-goat-anti-rabbit-igg-secondary-antibody

IRDye® 680RD Goat anti-Mouse IgG Secondary Antibody: https://www.licor.com/bio/reagents/irdye-680rd-goat-anti-mouse-igg-secondary-antibody

For IHC or Multiplexing
Anti-Cytokeratin 19, clone TROMA-3, Cat. No. MABT91: https://www.emdmillipore.com/US/en/product/Anti-Cytokeratin-19-Antibody-clone-TROMA-3,MM_NF-MABT913

Mouse PDGF R alpha Antibody, AF1062: https://www.rndsystems.com/products/mouse-pdgf-ralpha-antibody_af1062

Podoplanin Monoclonal Antibody (NZ-1.3), eBioscience™: https://www.thermofisher.com/antibody/product/Podoplanin-Antibody-clone-NZ-1-3-Monoclonal/14-9381-82

Anti-alpha smooth muscle Actin antibody (ab5694): https://www.abcam.com/products/primary-antibodies/alpha-smooth-muscle-actin-antibody-ab5694.html

Secondary HRP Polymer; Vendor/Cat#:
Goat-on-Rodent HRP Polymer, Biocare GHP516H: https://biocare.net/product/goat-on-rodent-hrp-polymer/
Rat-HRP-Polymer 1-Step, Biocare BRR4016H: https://biocare.net/wp-content/uploads/4016.pdf
Peroxidase-conjugated Anti-Syrian Hamster IgG, Jackson Labs, 307-036-003: https://www.jacksonimmuno.com/catalog/products/307-036-003
Rabbit-on-Rodent HRP Polymer, Biocare RMR622L: https://biocare.net/wp-content/uploads/RMR622.pdf

OOPAL Fluor: https://www.akoyabio.com/
520, Akoya FP1487001KT
690, Akoya FP1497001KT
570, Akoya FP1488001KT
620, Akoya FP1495001KT

Eukaryotic cell lines

Policy information about cell lines and Sex and Gender in Research

Cell line source(s)	PaTu-8988T, PANC1, MiaPaCa2, and PaTu-8902 were obtained from ATCC or the DSMZ. HY15549 and HY19636 were derived from female KPC mice (p48-Cre+;KrasLSL-G12D/+;Trp53lox/+) that were fully backcrossed into a C57BL/6 background All cell lines were grown in 5% CO_2 and 37C°. All cell lines were cultured in DMEM (Corning 10-017-CV) with 10% fetal bovine serum (FBS) (Atlanta Biologicals S11550H) and 1% Penicillin/Streptomycin (Thermo, 15140122).<br><br>Sex of the primary murine PDAC cell lines used in this study:<br>HY19636: female<br>HY15549: female<br><br>Sex of patient-derived specimens:<br>NYU341: Female<br>NYU559:Male<br>NYU280: Male<br>NYU338: Male<br>NYU345: Male

Authentication	Cell lines were authenticated by periodic fingerprinting and visual inspection, and low passage cultures were carefully maintained in a central lab cell bank.
Mycoplasma contamination	Cells were tested routinely for mycoplasma contamination by PCR. All cell lines and organoids used in this study tested negative for mycoplasma.
Commonly misidentified lines (See ICLAC register)	No commonly misidentified cell lines were used.

Animals and other research organisms

Policy information about studies involving animals; ARRIVE guidelines recommended for reporting animal research, and Sex and Gender in Research

Laboratory animals	All animal studies were approved by the NYULMC IACUC (Institutional Animal Care and Use Committee), Protocols #IA16-00507. Female mice B6J (Taconic, C57BL/6J), athymic (Taconic, NCrNU), or NSG (Jackson Laboratory) used in this study were 8-10 weeks old and not involved in previous procedures. All mice were maintained in the animal facility of the New York University Grossman School of Medicine.
Wild animals	No wild animals were used in the study.
Reporting on sex	Sex of patient-derived specimens:<br>NYU341: Female<br>NYU559:Male<br>NYU280: Male<br>NYU338: Male<br>NYU345: Male<br><br>The mouse and human pancreatic cancer cell lines were derived from both male and females. All the mice for this study were female and sex was not a specified variable for analysis .
Field-collected samples	No field collected samples were used in the study.
Ethics oversight	All animal studies were approved by the NYULMC IACUC (Institutional Animal Care and Use Committee), Protocols #IA16-00507.

Note that full information on the approval of the study protocol must also be provided in the manuscript.

Flow Cytometry

Plots

Confirm that:

- [x] The axis labels state the marker and fluorochrome used (e.g. CD4-FITC).
- [x] The axis scales are clearly visible. Include numbers along axes only for bottom left plot of group (a 'group' is an analysis of identical markers).
- [x] All plots are contour plots with outliers or pseudocolor plots.
- [x] A numerical value for number of cells or percentage (with statistics) is provided.

Methodology

Sample preparation	HY19636 cells were treated with DON (25µM) for 24 hours. Then, cells were treated with CellROX™ Deep Red Reagent (Fischer, C10422) at a concentration of 5µM, incubated for 30 minutes at 37C as described in the manufacturer protocol. Cells were washed with FCM buffer (HBSS containing 1% FBS, 1 mM EDTA, and 10 mM HEPES). Dead cells were depleted by DAPI staining. Experiment was performance twice and data included in paper show a representative experiment. The data was displayed as Mean Fluorescent Intensity (MFI) of bright deep red fluorescence signal.
Instrument	Cells were analyzed on a BD LSR Fortessa or a BD LSR-II UV and analyzed by FlowJo software (FlowJo, LLC, version 10.4).
Software	FACSDiva software (BD Biosciences) was used to collect the data, and the data was analyzed by FlowJo software (FlowJo, LLC, version 10.4).
Cell population abundance	The purity of cells was confirmed by viability (DAPI negative) usually > 90%.
Gating strategy	For FACS, singlet cells were identified by forward and side scatter, viability (DAPI negative).

- [ ] Tick this box to confirm that a figure exemplifying the gating strategy is provided in the Supplementary Information.
