# Supplementary material for: Targeting pancreatic cancer metabolic dependencies through glutamine antagonism
Source: Nat Cancer. 2023 Oct 9;5(1):85–99. doi: 10.1038/s43018-023-00647-3 (PMC10824664; doi:10.1038/s43018-023-00647-3)

Figure 5g

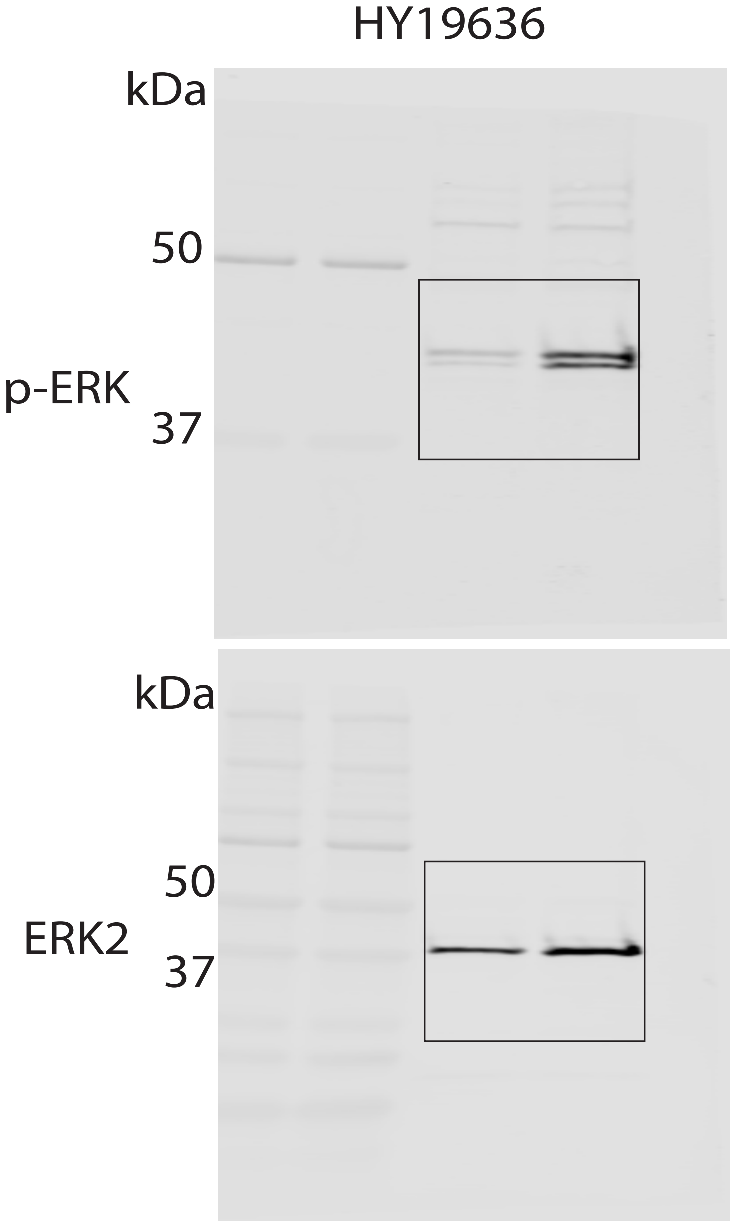

Image obtained through Licor.
p-ERK (CST 4376, 1:1,000)
ERK2 (sc-1647, 1:1,000)

IRDye® 800CW Goat anti-Rabbit IgG Secondary Antibody
IRDye® 680RD Goat anti-Mouse IgG Secondary Antibody

Figure 5g

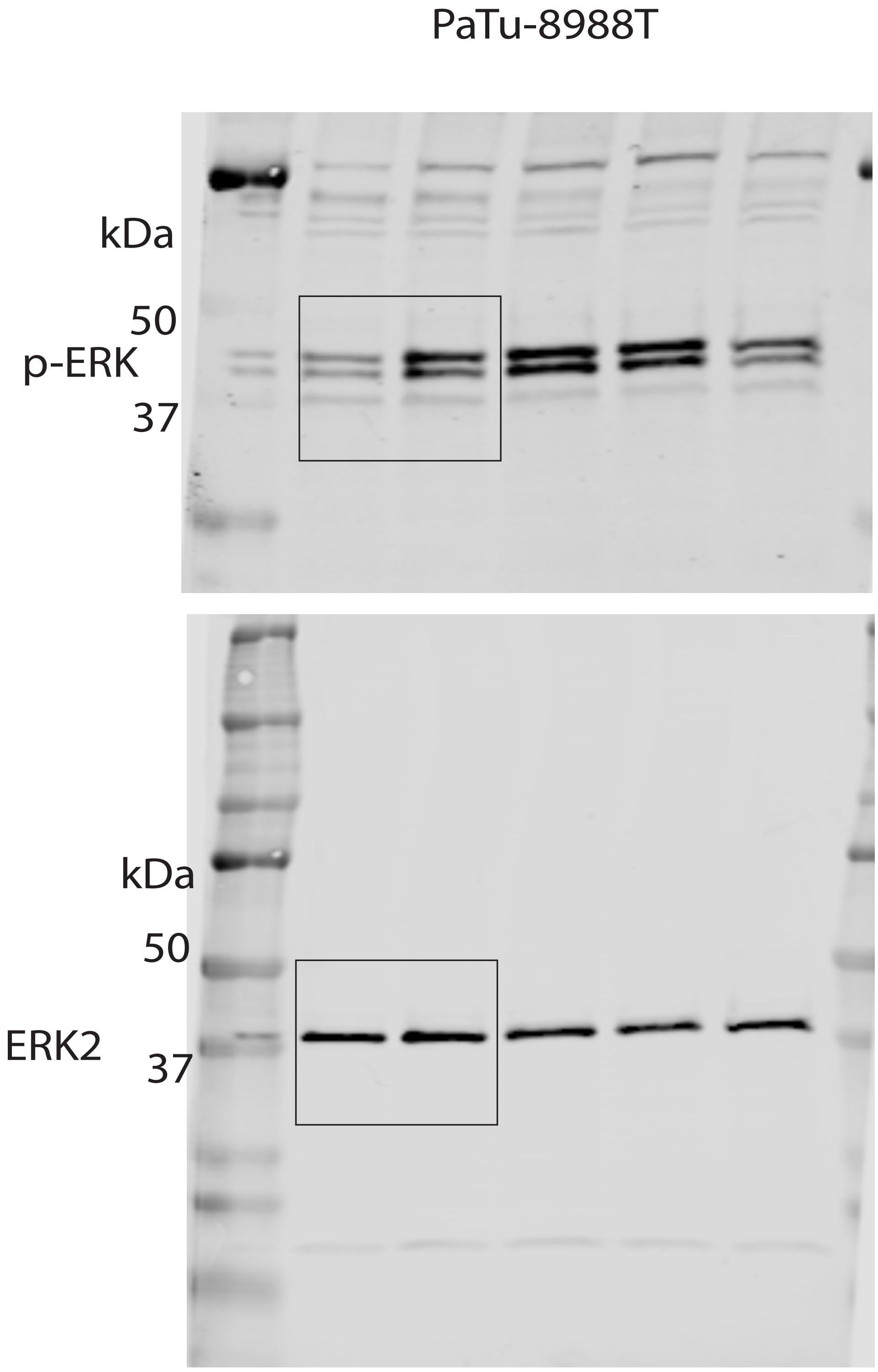

Image obtained through Licor.
p-ERK (CST 4376, 1:1,000)
ERK2 (sc-1647, 1:1,000)

IRDye® 800CW Goat anti-Rabbit IgG Secondary Antibody
IRDye® 680RD Goat anti-Mouse IgG Secondary Antibody

Figure 5g

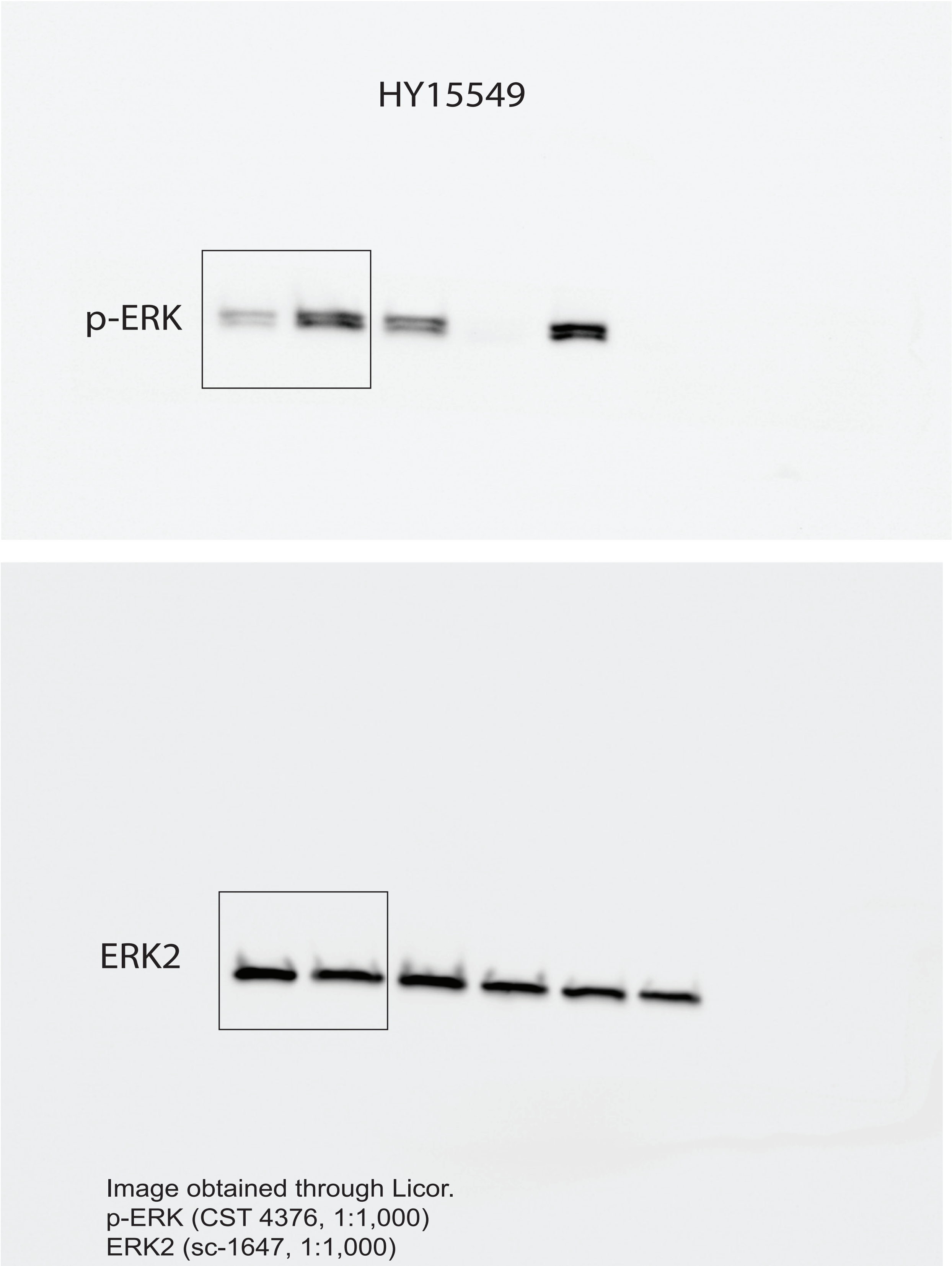

IRDye® 800CW Goat anti-Rabbit IgG Secondary Antibody
IRDye® 680RD Goat anti-Mouse IgG Secondary Antibody

Extended Fig 6c

Phospho-eIF2α (Ser51)
(CST, 3398, 1:1,000)

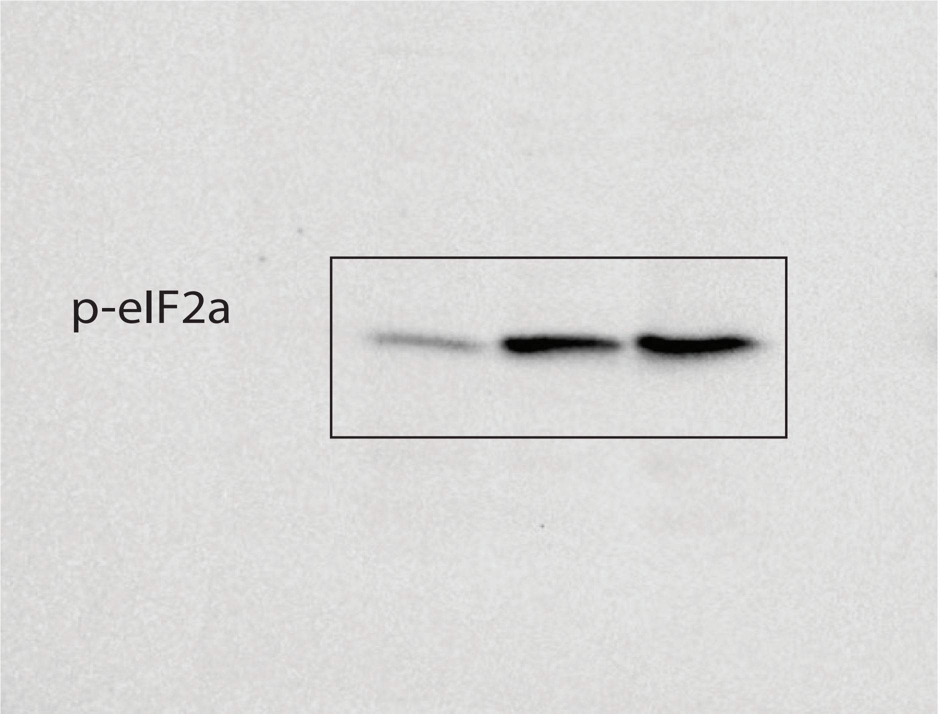

ATF4 (CST, 11815,
1:1,000)

ATF4

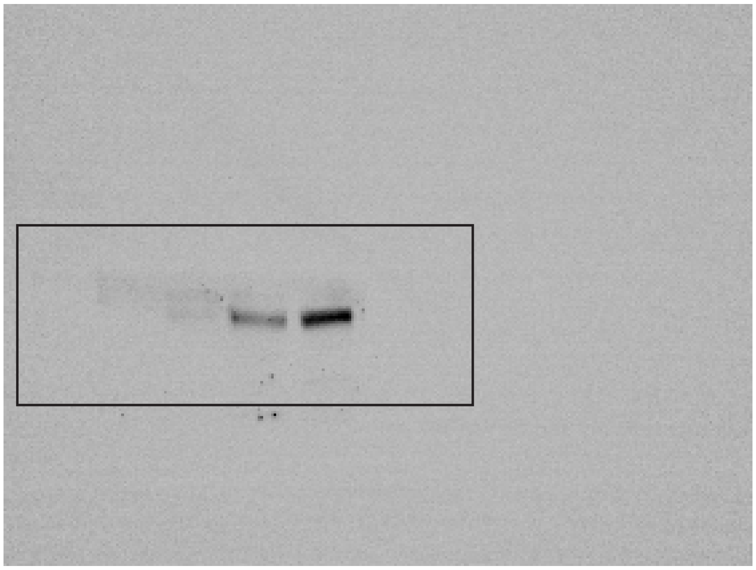

ERK2

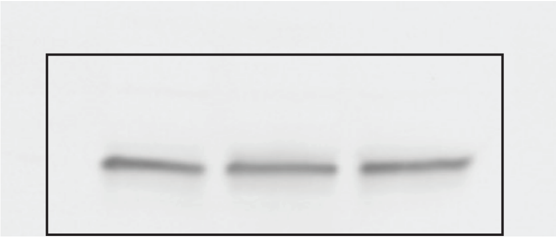

Extended Fig 6f

*

Anti-O-Linked
N-Acetylglucosamine antibody
[RL2](ab2739)

ERK2

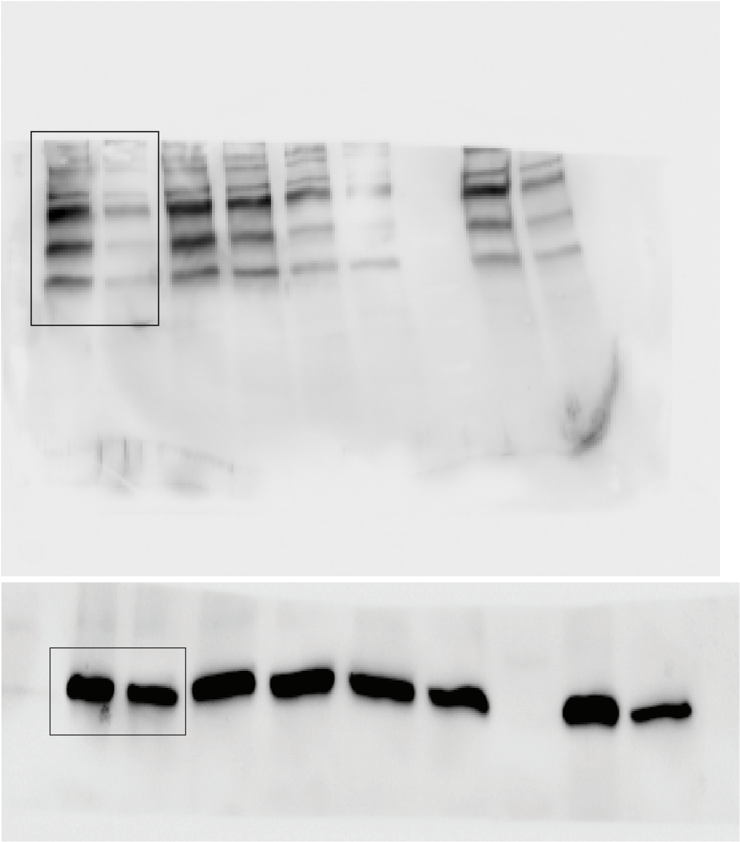

*

Same membrane as in Extended Figure 10e.
Gel was probed for p-ERK and ERK2, then reprobbed for
Anti-O-Linked N-Acetylglucosamine antibody

Extended Fig 9a

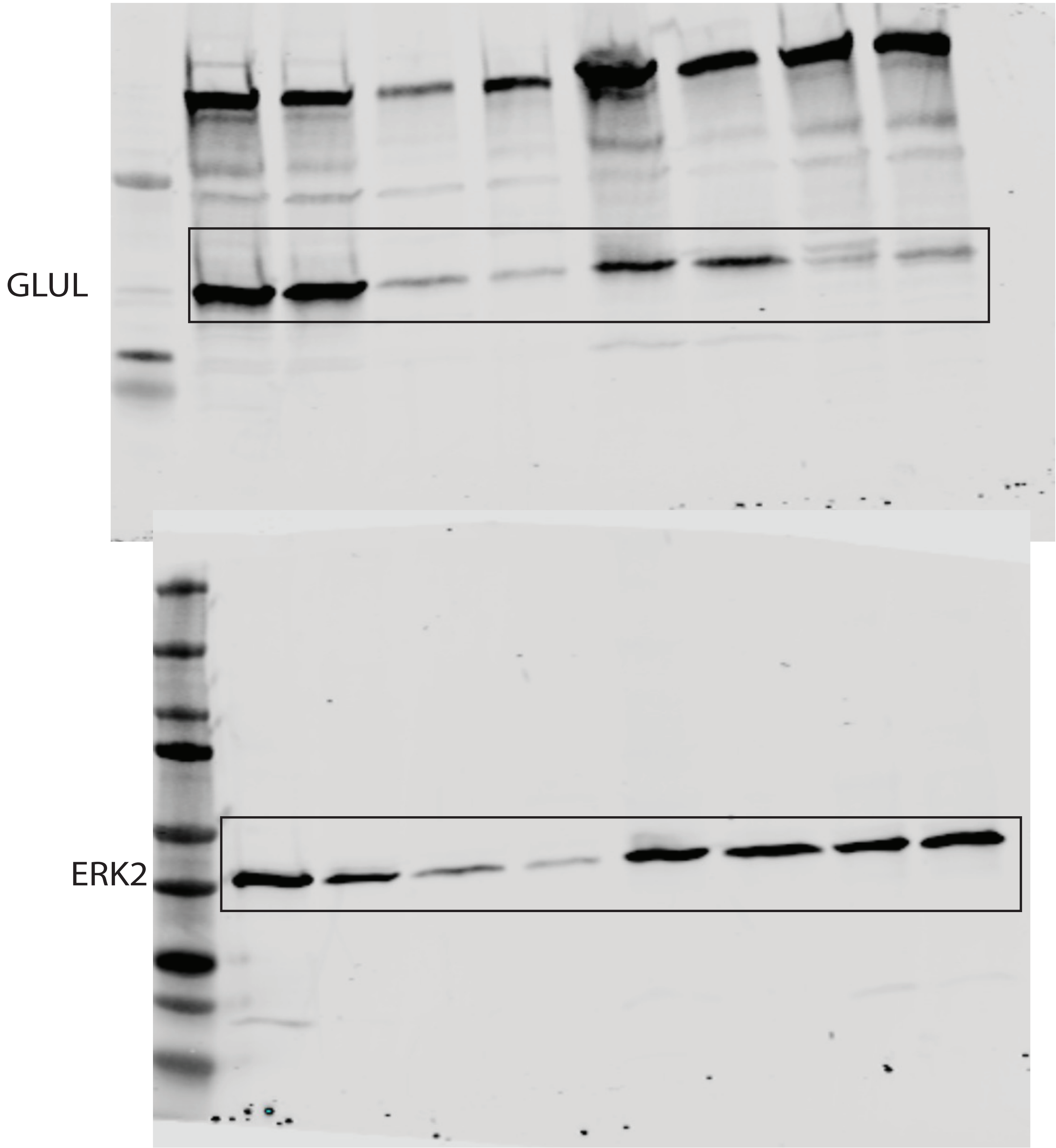

Image obtained through Licor.
GLUL (Abcam, ab228590)
ERK2 (sc-1647, 1:1,000)
IRDye® 800CW Goat anti-Rabbit IgG Secondary Antibody
IRDye® 680RD Goat anti-Mouse IgG Secondary Antibody

Extended Fig 9b

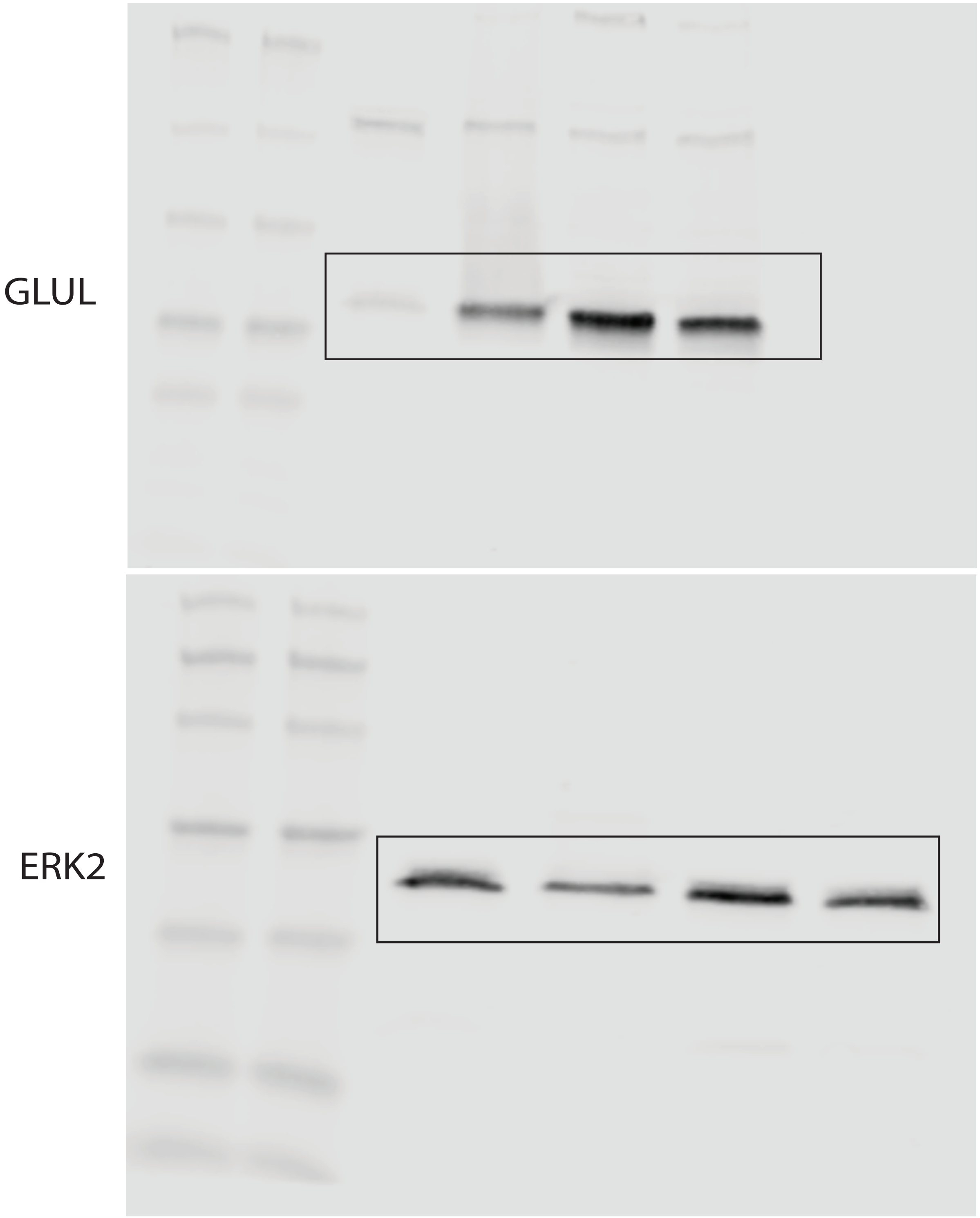

Image obtained through Licor.
GLUL (Abcam, ab228590)
ERK2 (sc-1647, 1:1,000)
IRDye® 800CW Goat anti-Rabbit IgG Secondary Antibody
IRDye® 680RD Goat anti-Mouse IgG Secondary Antibody

Extended Fig 10e

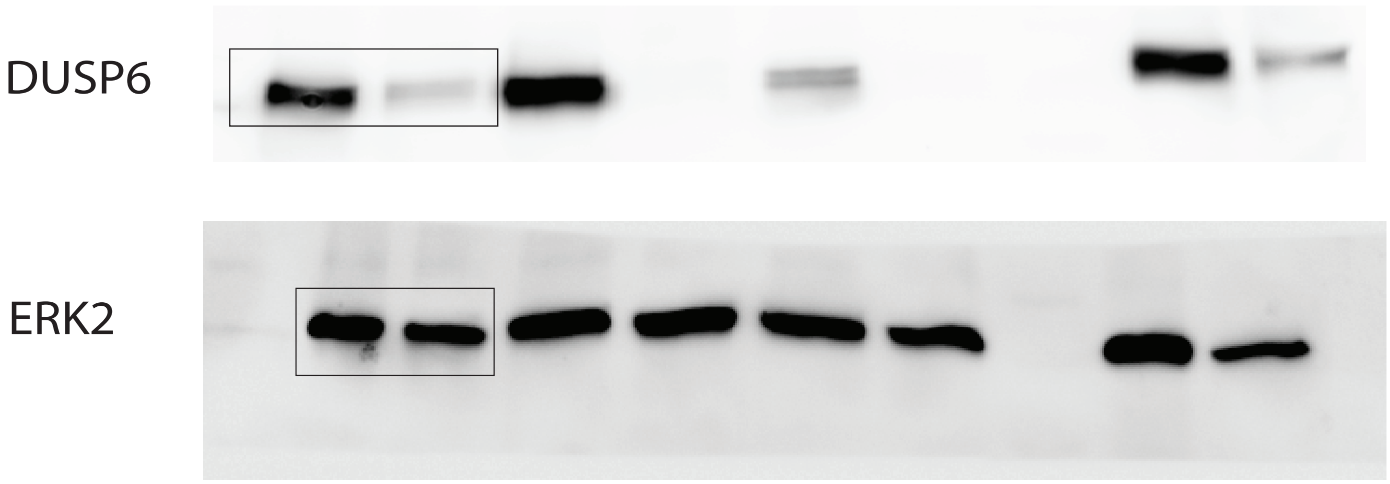

Image obtained through Licor.
DUSP6 (Abcam, A2D4, 1:1,000)
ERK2 (sc-1647, 1:1,000)

IRDye® 800CW Goat anti-Rabbit IgG Secondary Antibody
IRDye® 680RD Goat anti-Mouse IgG Secondary Antibody

Same membrane as in Extended Figure 6f.
Gel was probed for p-ERK and ERK2, then reprobbed for
Anti-O-Linked N-Acetylglucosamine antibody

Extended Fig 10f

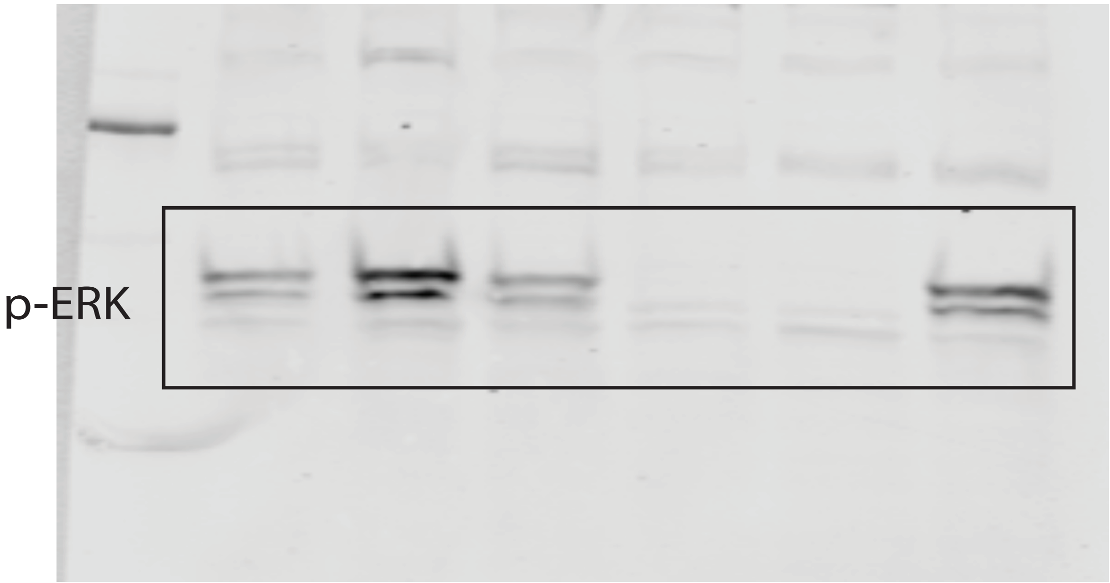

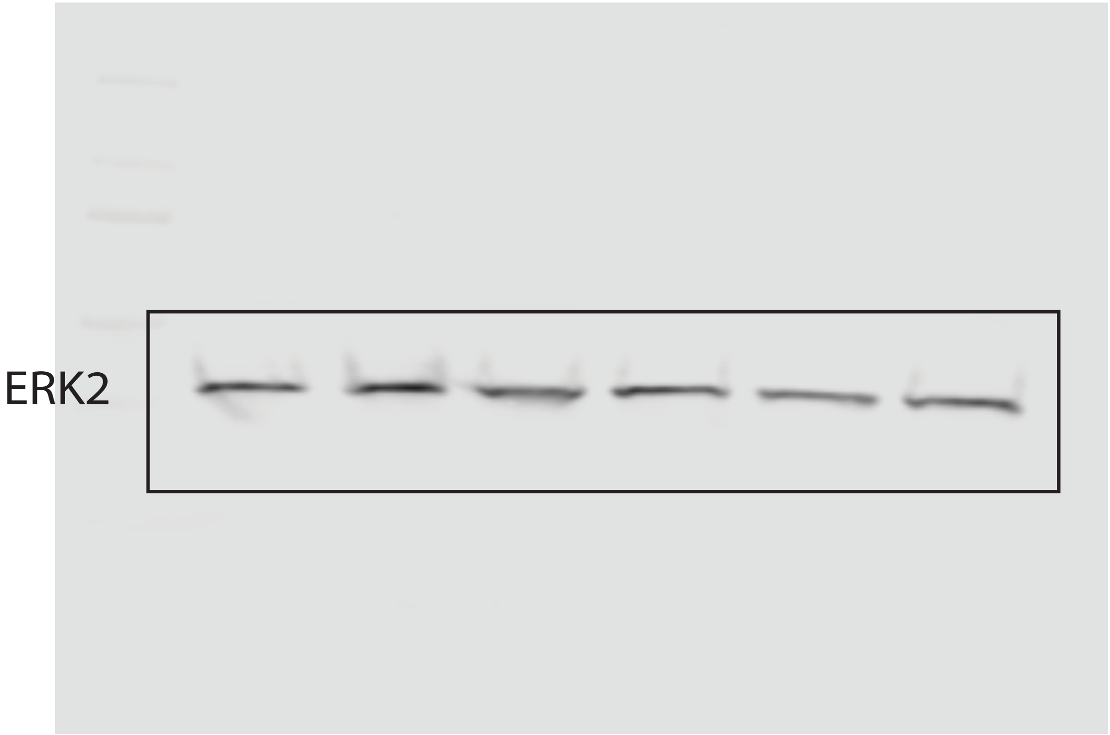

Extended Fig 10g

p-ERK

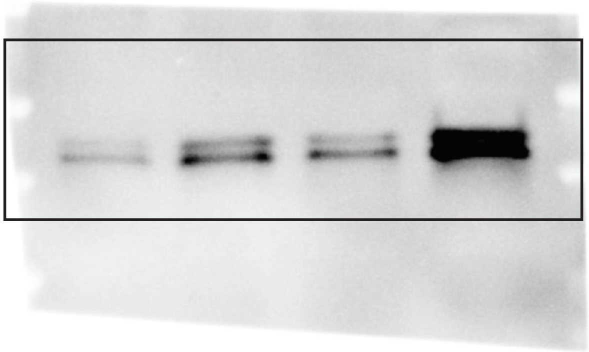

ERK2

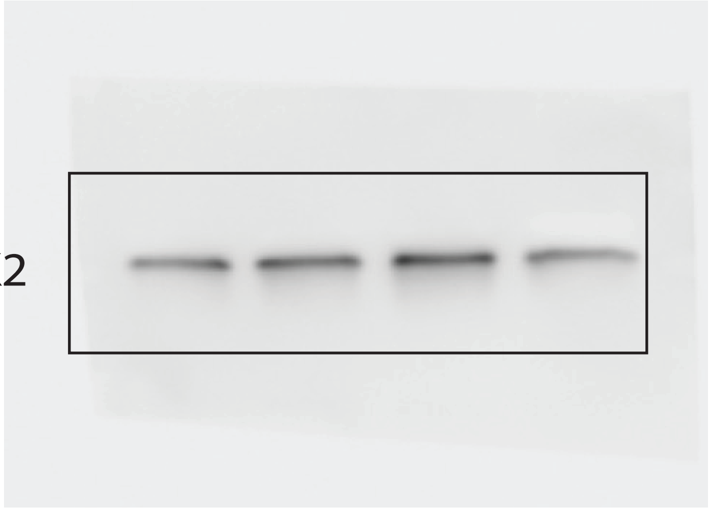

Image obtained through Licor.
p-ERK (CST 4376, 1:1,000)
ERK2 (sc-1647, 1:1,000)

IRDye® 800CW Goat anti-Rabbit IgG Secondary Antibody
IRDye® 680RD Goat anti-Mouse IgG Secondary Antibody

Extended Fig 10h

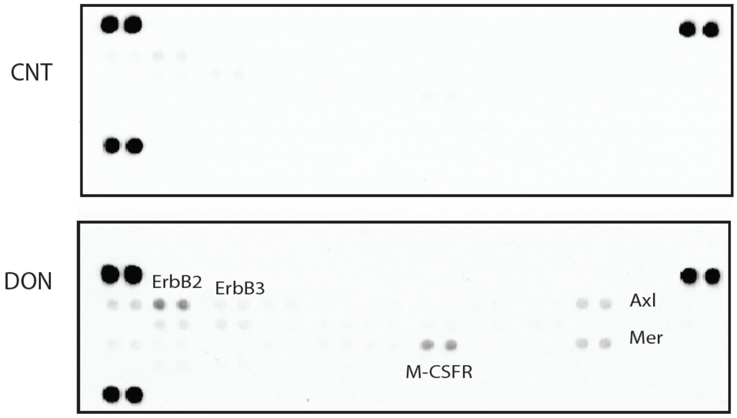

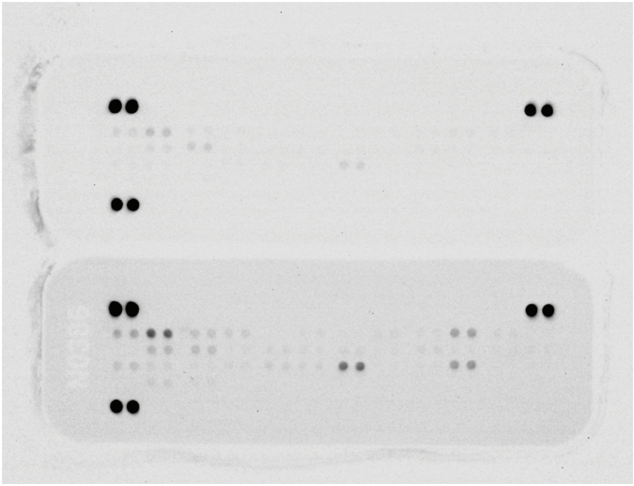

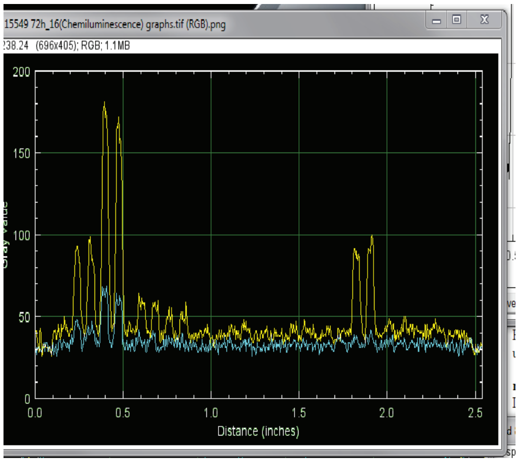

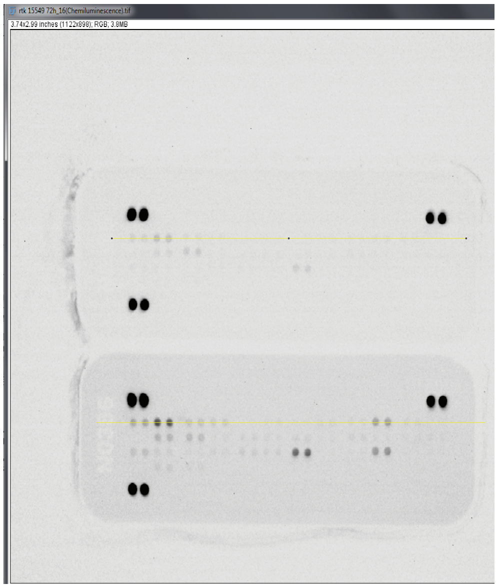

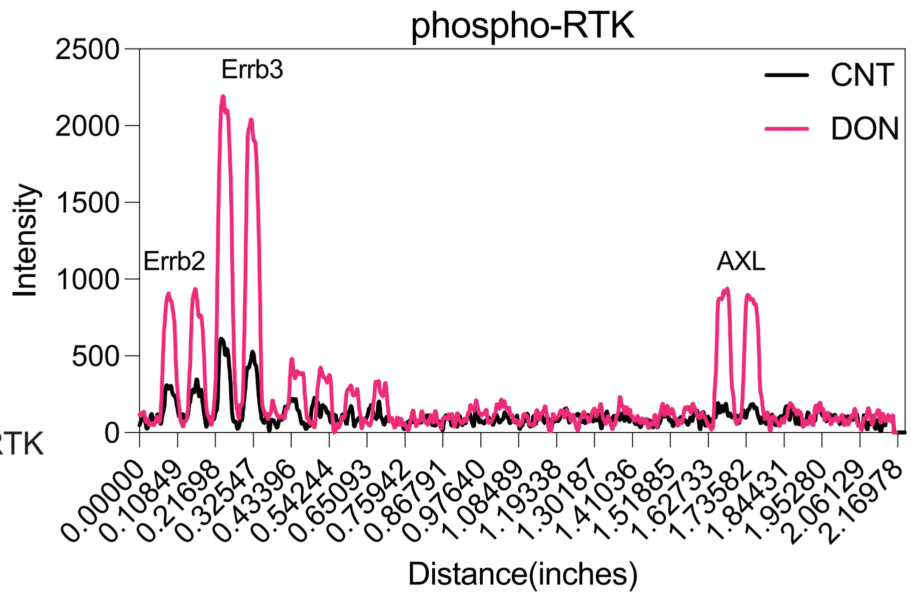

Image from Chemidoc
Analyze in ImageJ
Profiler Mouse Phospho-RTK
Array Kit (#ARY014)

Extended Fig 10i

PaTu-8988T
phospho-RTK

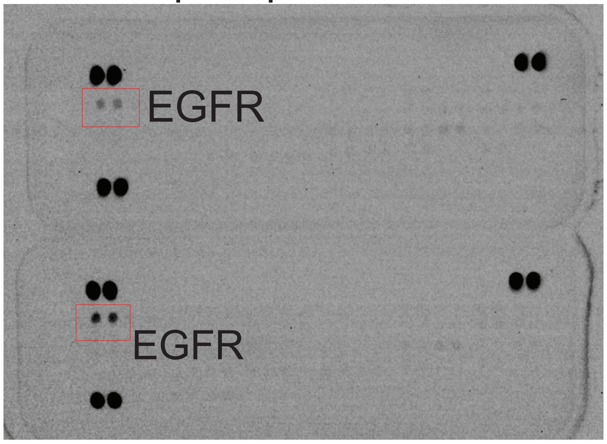

PaTu-8902
phospho-RTK

CNT

DON

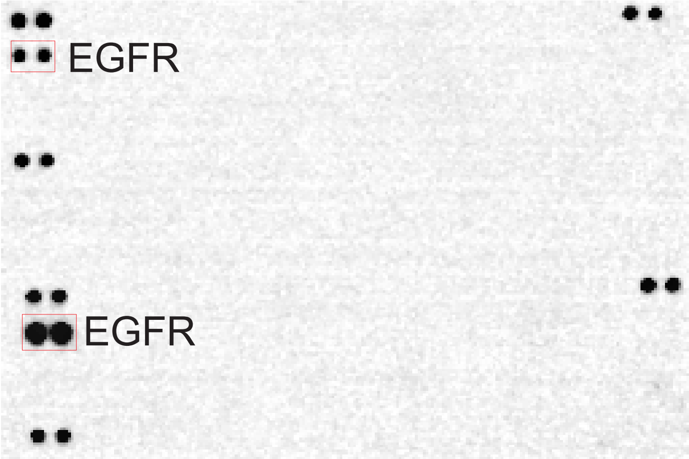

PANC1
phospho-RTK

CNT

DON

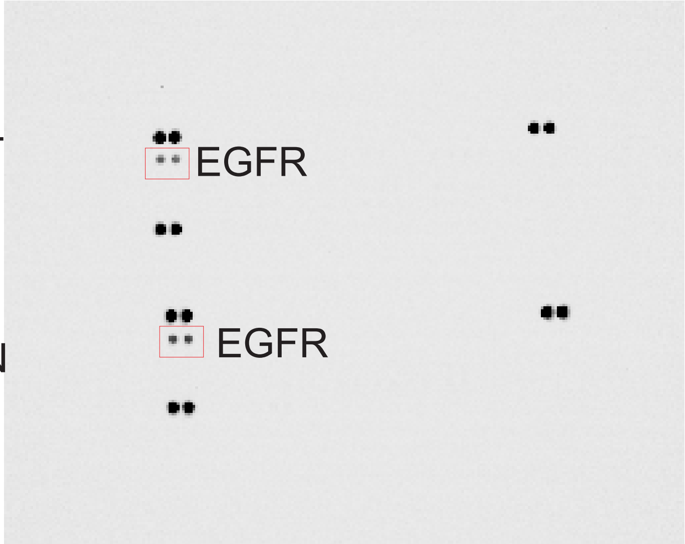

Image from Chemidoc
Analyze in ImageJ
Proteome Profiler Human Phospho-RTK Array Kit
(#ARY001B)

Extended Fig 10j

p-ERK

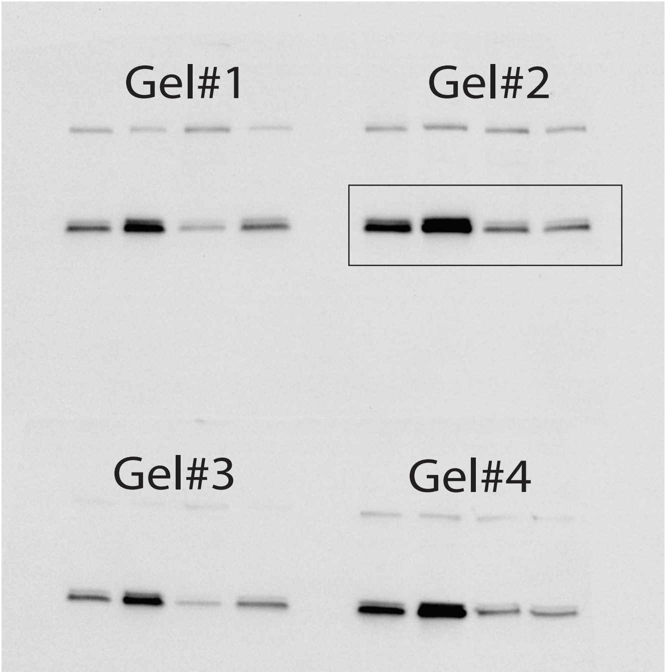

ERK2

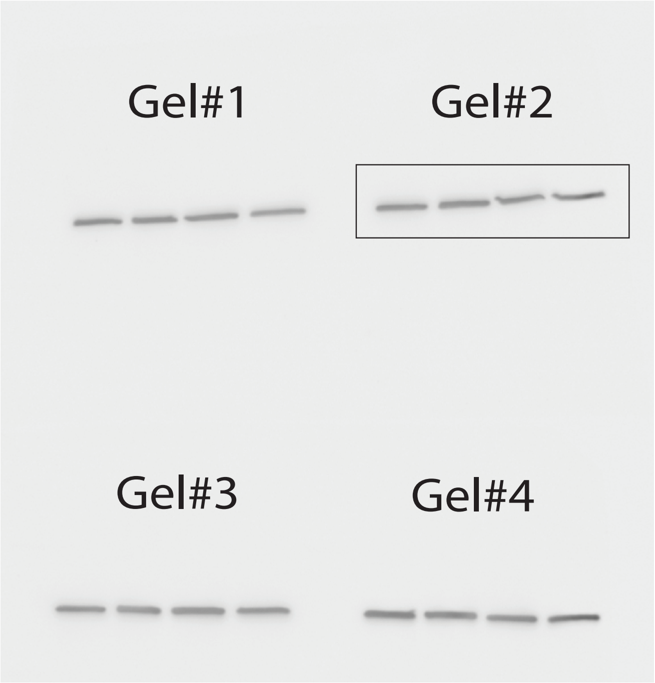

Extended Fig 10k

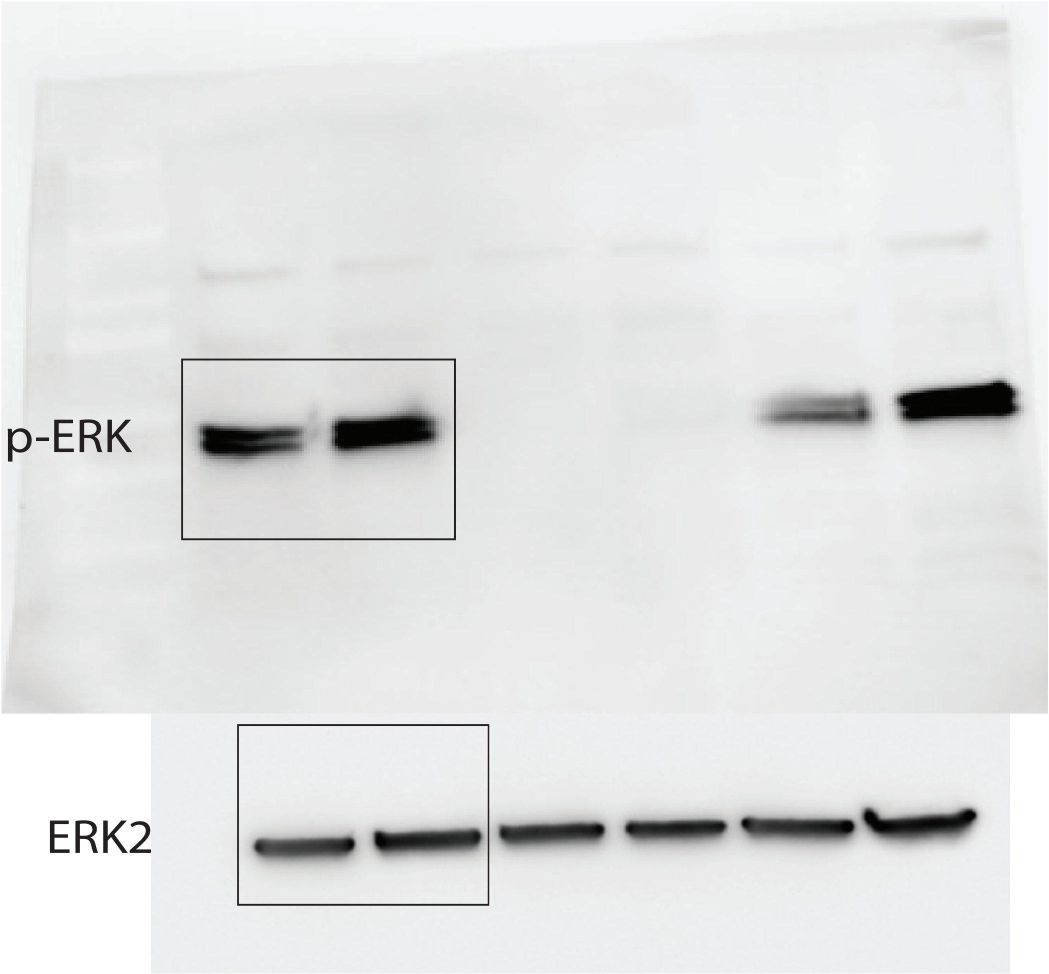

Supplement: Supplementary file 8 — Uncropped gels. [file 43018_2023_647_MOESM8_ESM.pdf]
